# Good Clinical Practice Guidance for Line Materials, Filtration, and Light Protection in Intravenous Medication Administration: Modified Delphi Consensus Study

**DOI:** 10.2196/88333

**Published:** 2026-06-10

**Authors:** Andrew Dickman, Penelope Tuffin, Rania Al-Jaber, Mona El-Harmeel, Jennifer Schneider, Irene Taladriz-Sender, Adam Sutherland, Robert Terkola, James Waterson

**Affiliations:** 1 Liverpool University Hospitals NHS Foundation Trust Liverpool United Kingdom; 2 Fiona Stanley Hospital Bethesda Hospital and Western Australia Country Health Service Australia; 3 King Faisal Specialist Hospital and Research Center Riyadh Saudi Arabia; 4 Children’s Cancer Hospital Cairo Egypt; 5 School of Medicine and Public Health Faculty of Medicine and Health Newcastle Australia; 6 Hospital General Universitario Gregorio Marañón Madrid Spain; 7 School of Pharmacy and Medical Sciences, University of Bradford Bradford United Kingdom; 8 Paracelsus Medical University Salzburg Austria; 9 Becton Dickinson and Company Dubai United Arab Emirates

**Keywords:** administration, consensus, panels, reviews, protection, panelists

## Abstract

**Background:**

There is an unmet need for reliable medication stability information to avoid suboptimal administration of intravenous medications, which may lead to reduced efficacy of therapy and potential patient harm through medication degradation or incompatibilities that cause vascular access issues.

**Objective:**

This study aimed to develop evidence-informed guidance for pharmacists and nurses on the use of administration line materials, in-line filtration, and light protection during storage and intravenous administration of medications commonly used in critical care and oncology.

**Methods:**

An initial list of 181 medications was compiled in consultation with pharmacist stakeholders from critical care and oncology specialties. A modified Delphi study was conducted over 3 rounds with a panel of 8 expert pharmacists selected for their clinical expertise, professional experience, and geographic location to ensure representation of diverse health care settings and medication administration practices. Panelists anonymously ranked statements on a 5-point Likert scale developed from a review of the literature with respect to the requirement for a specific administration line material for each medication; the need for, and type of, filtration required during administration; and the need for light protection during storage and administration of medications. After each round, items achieving 80% consensus were finalized. Those that did not achieve consensus were carried forward to the next round. This iterative approach allowed panelists to reconsider their ratings based on emerging group consensus and additional evidence shared by panelists between rounds.

**Results:**

A total of 1044 administration and storage requirements were assessed for a final list of 174 medications. In round 1, consensus was reached for 613 (58.7%) statements, with every statement being addressed and scored by the panelists. Panelists provided additional evidence sources for their decisions, and these were distributed to all panel members for round 2. All items were addressed and scored by the panelists. By the conclusion of round 3, consensus had been achieved for 697 (66.8%) statements, with all items being addressed and scored by the panelists.

**Conclusions:**

This study developed consensus-based recommendations for the selection of administration line materials, the use of in-line filtration, and light protection for the administration and storage of a range of medications administered intravenously. The guidance will aid medication stability and efficacy and promote good clinical practice; it currently underpins a prototype bedside app for correct intravenous administration line selection for nursing and pharmacy staff in critical care and oncology units.

## Introduction

Pharmacists are considered, by both medical and nursing staff, to be the arbiters of administration knowledge for intravenous (IV) medications. Knowledge of administration line material types and their associated requirements, including light protection and filtration, has been reported to be poor, even among nurses working in specialist disciplines [1]. This is despite approximately 40% of nursing time being devoted to medication administration [2]. In a survey of Spanish nurses, 80.8% of respondents reported having “a good level of knowledge on the preparation and administration of IV medicine,” although 88.1% also reacted positively to statements on the need to improve their skills [3].

While pharmaceutical manufacturers provide guidance on the storage and protection of parental medications, there is generally much less information available to the end user after reconstitution or following compounding processes. Furthermore, preparation of IV medicines takes place in many locations across facilities, and information should be provided for their mixing and administration across many medical specialties. Some health systems provide medications compounded in the pharmacy or by a commercial compounder, in which cases, the package inserts may be unavailable for consultation by the nursing staff who administer the medication. Conversely, in many health systems, most IV medication preparation is undertaken by nurses in the clinical area without direct pharmacist support [4], and often package inserts or supporting guidelines do not offer information on storage and handling after reconstitution. For example, there may be an assumption that medicines prepared on the ward will be administered immediately, but this is not always the case.

An extensive survey of sterile compounding errors in American hospital pharmacies indicated that wrong preparation techniques, including the attachment of an incorrect administration line, were identified in 26% of compounding errors [5]. A key contributory factor to these findings was the lack of standardized processes. The need for systems-focused interventions, including standardized pathways, has been recognized as a key concept in driving efficiency and sustainability in cancer care [6]. A substantial component of such tools would be dedicated to improved compounding processes, final-product preparation, labeling, and the provision of information [7].

There are many variables to consider when developing standard practice guidelines for medication preparation and administration, and these differ depending on where and how the medication is prepared for administration. However, with the assumption that most preparation occurs at the bedside and that, once a medication is reconstituted or diluted, members of the health care team need a practical reference guide for IV medication stability, the requirements for such a clinical reference fall broadly into the following 3 categories: the need for filtration, the need for protection from light during storage and administration, and the material composition of administration systems—syringes, bags, and lines. These factors are essential for maintaining medication stability and effectiveness, yet this information is often missing from standard drug references.

Additionally, there are 3 potential medication-material interactions: absorption into the administration line, adsorption onto the administration line, and leaching or migration of materials into medication solutions. Reduced efficacy of medications due to product degradation is a concern because it has the potential to directly impact patient care and management and affect treatment outcomes associated with regimens and individual medications. Vascular access issues, patient condition, or delayed laboratory results can cause delays in medication administration in both critical care and oncology treatment centers [8].

Such delays may be important in terms of the integrity of the compounded solution if it is sensitive to light exposure or affected by contact with administration line material.

The deleterious effect of light on some compounds, such as parenteral nutrition, is well known [9], but the effect of light on other medications is less well established. Approximately 30% of the degradation of clear medication solutions due to light may occur in the line or tubing of the infusion set [10], and this would logically increase at very low flow rates.

There is also an aspect of pharmaceutical waste in these circumstances, whereby medications that are not administered may be discarded because of uncertainty regarding their stability outside the controlled environments in which they are stored and prepared.

There is an unmet need for reliable and unambiguous medication stability information to support clinical decision-making at the bedside. This study aimed to develop guidance for different types of administration lines, in-line filtration, and light protection during storage and administration through a review by subject matter experts of available evidence for 181 IV medications commonly used in critical care and oncology.

## Methods

### Overview

The stability of IV admixtures is fairly well studied; however, the literature on this topic is vast and often contradictory. Therefore, we sought expert consensus to inform pragmatic information for clinicians and conducted a 3-round Delphi study. The Delphi approach is considered a powerful method that can deliver agreement in areas of uncertainty or where definitive evidence is lacking, and it allows expert opinion to emerge through consensus [11-13].

We formed a panel of 8 subject matter experts, identified for their professional experience, geographic location, patient population served, and compounding processes and workflows used in practice. The panel represented practice and expertise from Europe, the United Kingdom, and Australia, and practice in hospitals, manufacturing units, and academia. The panel also represented pediatric practice, critical care, oncology and palliative care. The panel members were selected by the lead author (AD) and study facilitator (JW), but the panel was otherwise anonymous. Initial agreement to participate was secured by email exchange and a formal letter of commitment. AD acted as chair of the panel and oversaw data analysis and the mitigation of disagreements in later stages.

Our modified Delphi process ran over 3 rounds. After 2 rounds, a further review was conducted by panel members who were considered outliers with respect to consensus. They were shown the anonymized scores of other panel members who had reached consensus. In addition, the panel chairperson (AD) and cochairperson (PT) reviewed these results and had casting votes. The reason for this approach was that, while local practice was actively sought as a constructive contribution to the Delphi panel, it might also act as an anomaly if practitioners had not been exposed to recent evidence regarding an individual medication. In this way, it was hoped that the Delphi panel process would itself facilitate consensus by sharing knowledge and challenging assumptions. Outlier selections by only one panel member were subject to the casting votes of the chair and cochair, but, as noted later, this only extended to exposing the panel member to the scores of other (still anonymized) Delphi panel members and encouraging further review of their selection. This modification of the Delphi technique is in line with current thinking on the process, which advocates “controlled feedback” [14].

Panel members were presented with an evidence dossier of 181 IV medications (presented by their recommended international nonproprietary names) and a library of literature to support assessment and opinion development. The following references were used to build the dossier:

1. Stabilis.org (independent pharmacy advice system) [15]
2. Medicines and Light, Oslo University [10]
3. University of Illinois at Chicago College of Pharmacy, Drug Information Group, with the 2023 update used upon publication [16,17]
4. Manufacturer package inserts
5. Individual independent scientific studies addressing specific medications and research on material compatibility, filtration, and light protection requirements

Sources 1 to 3 were selected because they were either recently published or updated frequently. Source 4 was selected as these are the evidence presented to and accepted by regulatory bodies. The independent scientific studies (n=5) were obtained via individual medication name searches undertaken in PubMed, Google Scholar, and under searches using specific search terms related to administration line materials. Evidence sources were evaluated according to time since publication and the strength of their conclusions. Where multiple sources were available, literature that most closely met the above criteria was selected. A sample of the evidence dossier is given in Multimedia Appendix 1.

Delphi panel members were instructed to work independently to determine their own opinions regarding the administration requirements of each medication with regard to material selection of the administration line, the need for protection from light during storage and administration, and the need for filtration of the product during administration. They were advised to use real-world assumptions to guide their deliberations: there may be delays between compounding and administration due to compounding processes, such as batching and preparation ahead of time, and patient-related factors (such as vascular access issues) could lead to an extended period during which the infusion would be exposed to light and possible administration line material interactions.

The threshold for consensus across the 8 panel members was set at 80% for each question asked. After each round of questions, those medications and their administration characteristics that reached 80% consensus were removed from further rounds. In the third (and final) round, the chair approached those panelists who were outliers in their responses. These panelists were shown the anonymized decisions of other panel members and asked whether they wished to reconsider their responses. Four medication profiles were resolved using this process, with 1 panelist per medication (3 individual panelists overall) being identified as an outlier.

The role of the chairperson was to oversee data analysis, identify and remove items that had reached consensus at each round, and field comments, questions, and queries from panel members. Panel members were also encouraged to identify and present new evidence if, within their scope of experience, they felt it was relevant, but the chairperson made decisions on the inclusion of this new data.

### Ethical Considerations

Given the absence of human participants or their data and the noncontentious nature of the questions being asked, the study was undertaken as a service evaluation. All variables in the worksheets were vendor-neutral. Panel members received reimbursement for their time. Individual participation by panel members was undertaken under the supervision of the Ethics and Compliance Department of Becton, Dickinson and Company and according to the Becton Dickinson Global Standards for Interactions with Healthcare Professionals, Healthcare Organizations, and Government Officials (effective March 1, 2022) and the Becton Dickinson Global Interactions with Healthcare Professionals Policy (effective October 1, 2019). Reimbursement and the expected duration of the work involved were agreed upon through four standard operating procedures for engagement of health care professionals according to this policy:

1. Calculation of a reimbursement fair market value (hourly rate) according to profession, qualification, standing, and geography
2. Cosigning of a master services agreement stating the fair market value and general conditions of engagement
3. A cosigned statement of work stating the task to be undertaken and the expected duration of the activity
4. A cosigned privacy agreement stating the rights of panelists regarding data protection under the General Data Protection Regulation: Regulation (European Union) 2016-679 of the European Parliament and of the Council of April 27, 2016

### Delphi Procedure

Panelists were provided with 3 worksheets created in Microsoft Excel (version 17). Each worksheet considered a different aspect of the administration or storage of the medication—material of the final administration line, protection from light, or the need for terminal filtration at the point of administration. Panelists were presented with recommendations from the evidence dossier. Because there was likely to be variation in practice and experience, and because for many medicines, answers to the questions could be ambiguous (eg, morphine could be refrigerated or not with no impact on the stability of the end product), a binary “Yes/No” response would not have captured those medicines for which storage or administration materials were critical and those for which these factors were less important. Therefore, panelists were asked to rate the strength of their decision on a 5-point Likert-type scale. This scale ranged from 1 (“strongly disagree”) to 5 (“strongly agree”). A score of 3 indicated no strong opinion. We chose a 5-point Likert scale because there is no absolute definition for the tools a Delphi panel should use, and we were interested in identifying disagreement as much as consensus. However, it was important to give panelists distinct and clear statements for evaluation [11,17,18].

### Definition of Consensus

Classically, Delphi panel methods accept consensus at ≥70% for individual-item removal from subsequent rounds [18]. This is commonly because there is a dearth of empirical evidence in areas where the technique is applied [19]. Given the importance of appropriate administration practice, the accepted threshold for consensus in this study was set at 80%. Likert weighting was used as the first point of assessment of the overall panel response. This required median scores of 1 to 2 (negative) or 4 to 5 (positive) on the Likert scale to be considered a collectively valid response to the statement being reviewed. The degree of consensus was calculated using the SD of the panel members’ scores. Percentage consensus was then derived using the Excel formula:

Consensus percentage=(1–SD/2)×100

In practice, it was found that, for items where the median did not reflect a strong opinion (median >2 and median <4), the SD was also always >0.4, giving a consensus score of less than 80%.

After the first round, items with ≥80% consensus were removed from review. Comments from panel members in round 1 were reviewed by the chairperson and cochairperson, which led to the removal of several medications. For example, moxetumomab was deleted based on its removal from the European market in 2021 and from the US market in 2023 [20].

Panel members also offered further evidence sources for their score selection in round 1. The chairperson and cochairperson added these to the second-round review. Therefore, the evidence base grew and could alter opinions and gain more consensus through subsequent rounds. For the remaining items, each member’s scores were returned to them in a workbook with instructions to review them as in round 1, and to apply any change they felt was appropriate to their score.

After round 2, items with ≥80% consensus were removed from further analysis. Items were identified for which a maximum of 2 panel members’ determinations were outside the consensus (ie, outliers). Mini-review sheets were created for individual panel members whose scores for individual medications were outliers, and all panel members’ scores (aggregated while maintaining anonymity) were revealed to the outlier panel member. The chairperson and cochairperson also rated these medications, effectively using their casting votes for these medications only.

After this final round of review, the process was closed, with items with >80% consensus being carried forward as clinical end-user guidance.

## Results

Following the removal of medications no longer marketed, 174 medications were assessed. Of the 1044 IV medication administration requirements assessed, there was ≥80% consensus for 613 (58.71%) items after round 1, rising to 697 (66.76%) items after 3 rounds. The number of definitive and nondefinitive recommendations achieving ≥80% consensus for each attribute assessed is shown in Table 1.

**Table 1:** Number of definitive and nondefinitive recommendations (N=1044)^a^.

Recommendation category			Definitive recommendations (≥80% consensus; n=697), n (%)		Nondefinitive recommendations (<80% consensus; n=347), n (%)
Di(2-ethylhexyl) phthalate–free polyvinyl chloride administration set			132 (75.86)		42 (24.14)
Polyethylene administration set			123 (70.69)		51 (29.31)
Polypropylene administration set			93 (53.45)		81 (46.55)
**In-line filtration**					
	0.2-μm filter	16 (9.20)		10 (5.75)	
	1.2-μm filter	2 (1.15)		1 (0.57)	
	0.2- to 1.2-μm filter	1 (0.57)		2 (1.15)	
	0.2- to 5-μm filter	1 (0.57)		0 (0)	
	15-μm filter	0 (0)		2 (1.15)	
	No filter required	131 (75.29)		8 (4.60)	
**Light protection during storage**					
	Yes	76 (43.68)		41 (23.57)	
	Not required	26 (14.94)		31 (17.81)	
**Light protection during administration**					
	Yes	8 (4.60)		62 (35.64)	
	Not required	88 (50.59)		16 (9.17)	

^a^For full output by each assessed medication, refer to Multimedia Appendix 2.

Table 2 illustrates how consensus and no-consensus conditions were determined. Some consensus issues were due to highly conflicting (and sometimes emerging) evidence. This highlighted how the final consensus opinion could diverge strongly from the initial evidence statement presented to the panelists for review. This was most commonly seen in the sections related to light protection during administration and filtration.

**Table 2:** Medication evidence statements with panelist comments and consensus alignment from rounds 1 and 2 of the Delphi panel.

Presented statements		Medications	Sample panelist comments	Consensus alignment (%)
**Light protection during administration**				
	**Yes**			
		Temsirolimus (Torisel)	“reference 234”; “torisel should be protected from excessive room light and sunlight”	100.0
		Leucovorin (calcium folinate)	“injectable drugs guide protect from light”; “however AHFS [American Hospital Formulary Service]: stable for 24 h when at RT^a^, protected from light”; “however evidence that reconstituted solutions of drug are not adversely affected by exposure to room light (Calcium leucovorin)”	61.8
		Filgrastim	“no mention...Injectable drugs guide...also stabilis studies under light and reference 275: diluted neupogen can be stored at RT for up to 24h, no mention of protect from light”	39.3
		Necitumumab	“Store protected from light. Brief exposure to ambient light is acceptable while preparation and administration is taking place. If delay in admin, protect from light. Score=4 to err on side of caution”	39.3
		Decitabine	“this is not stable drug- no mention of light in monographs but 1 h room temp. Within 15 minutes of reconstitution, the concentrate (in 10 ml of sterile water for injections) must be further diluted with cold (2 °C-8 °C) infusion fluids. This prepared diluted solution for intravenous infusion can be stored at 2 °C-8 °C for up to a maximum of 3 hours, followed by up to 1 hour at room temperature (20 °C-25 °C) before administration.”	37.1
		Naxitamab-gqgk	“protect until time of use. FDA advice per document 2020/761171	31.3
		Daunorubicin	“reference 5”	29.3
		Bendamustine	“ref 5: stability of diluted solutions affected by light”; “the final admixture is stable for 24 hours when stored refrigerated (2-8 °C or 36-47 °F) or for 3 hours when stored at room temperature (15-30 °C or 59-86 °F) and room light. Administration of TREANDA must be completed within this period”; “source:https://www.accessdata.fda.gov/drugsatfda_docs/label/2008/022303lbl.pdf. (err on side of caution due to 3h)”	26.9
		Chlorphenamine	“no reference, usually give over short time: use immediately after dilution, so protect from light not relevant Administered as a short infusion/bolus so wouldn't be unwrapped until the moment of use usually [AS]”	25.5
		Cisplatin	“Cisplatin 0.15 mg/mL in sodium chloride IV infusion 0.9% is chemically stable for 24 hours when stored at room temperature and protected from light. https://medsinfo.com.au/product-information/document/DBL_Cisplatin_Injection_PI. Also Baertschi reference”	25.5
		Doxorubicin liposomal	“no specific reference but because of doxorubicin component, err on side of caution”	25.5
		Bevacizumab	“No information stating need light protection during admin”	23.6
		Bortezomib	“reference 5 states reconstituted solutions affected by light but Velcade states does not need to be protected from light when admin: also not longer than 8h when exposed to room light (change from 5 to 3)”	19.6
		Trastuzumab emtansine	“infusions over 30 to 90 min; only information I can find says if not immediately administer, store in refrigerator; refrigerators may or not protect from light…”	16.3
		Digoxin	“no reference states protect from light Administered as a short infusion/bolus so wouldn't be unwrapped until the moment of use usually”	10.2
		Meropenem	“no reference to light. Injectable drugs guide Administered as a short infusion/bolus so wouldn't be unwrapped until the moment of use usually”	10.2
		Amiodarone hydrochloride	“Illinois Chicago reference states does not need to be protected from light during admin BUT avoid exposure to direct sunlight (3 because of sunlight reference): however Tonnesen reference refers to amiodarone as light sensitive critical care drug”	9.9
		Midazolam hydrochloride	“no reference to protect from light injectable drugs guide. No outcome related data to support this”	5.7
	**Not required**			
		Daratumumab	“reference mentions followed by 15 h including infusion time at RT and room light”	25.5
		Daunorubicin liposomal	“based on daunorubicin protected from light, erring on side of caution would say protect this formulation from light”	25.5
		Dexamethasone	“given over short time analyzed it myself”	25.5
		Etoposide	“micromedex stability studies indicating stability under fluorescent light”	25.5
**Filtration no or filter size during administration**				
	1.2	Infliximab	“reference Hospital Pharmacy Ipema et al”	81.4
	0.2	Tagraxofusp	“reference 233: elzonris refers to using 0.2-μm filter”	81.4
	0.2	Avelumab	“Aust Inject Handbook and also reference 29 state use 0.2-μm low protein binding filter”	62.7
	Not required	Atezolizumab	“reference 26: use of in-line filter membranes is optional”	61.8
	1.2	Asparaginase (*Escherichia coli*)	“reference mentions use of 5-μm filter”; “loss of potency with 0.2 μm”; “this spreadsheet states 1.2 μm-perhaps better to be 5 μm?”; “also, this product may no longer be available? Changed to 4 due to filter size”	55.1
	Not required	Asparaginase Erwinia	“no mention of use of filter score remains at 5”	39.3
	0.2	Cemiplimab	“reference Hospital Pharmacy Ipema et al”	26.9
	0.2	Dinutuximab	“reference 103 recommended that a 0.22-μm in-line filter during infusion”	26.9
	0.2	Blinatumomab	“product information states to use 0.2-μm filter (reference 263) ipema et al Hospital Pharmacy (7 day not required)”	25.5
	Not required	L-asparaginase	“filter may be used if fibrous particles 5 μm and no loss of potency, some loss of potency with 0.2 μm ref 154”	25.5
	Not required	Moxetumomab	“no reference to use of filter”	25.5
	0.2	Magnesium sulphate	“If diluted below super-saturated solution (50%) then no filter required in practice”	23.6
	0.2	Clofarabine	“SPC [Summary of Product Characteristics]: Clofarabine-ratio pharm 1 mg/ml Concentrate for Solution for Infusion be filtered through a sterile 0.2-micron syringe filter and then diluted with a 9 mg/ml (0.9%) sodium chloride infusion solution)”	16.3

^a^RT: room temperature.

## Discussion

### Principal Findings

We have generated an extensive list of medications, with recommended administration sets and storage and handling requirements, that can be applied at the bedside to potentially improve access to high-quality medication information. The Delphi panel method proved to be a useful tool for creating consensus-based guidance for clinicians administering IV medications, many of which might be considered specialized therapies. The study goal was achieved by creating a distilled and easy-to-understand guide. The guidance is comprehensive for the parameters reviewed, and its basis in published evidence and professional expertise should provide clinicians with confidence in any tool within which it is subsequently deployed. Furthermore, by distilling a diverse range of sources into a single output, we potentially mitigate some of the idiosyncrasies commonly present in localized clinician assessments.

There is potential for this tool to reduce avoidable medication waste and unnecessary storage, which supports health care initiatives aimed at improving sustainability and efficiency. This is additionally important given the financial burden of medication waste on health care systems and patients [19].

The panel made a collaborative decision during a final open meeting on the required frequency of review of the medication list, based on likely changes in the evidence base, new administration line materials, and new medications entering the market. This was set at 24 months, which is within the review timing of other medication review sites, such as the National Injectable Medicines Guide in the United Kingdom [21]. The assessment of new medications, either requiring an interim decision between formal reviews or addition to the “pending” list for future panel review, is possible based on local experience and the review of dashboard sites, such as the UK National Institute for Health Research Innovation Observatory [22].

### Limitations

There are limitations to the study. Only the most common administration line materials and those most readily available to clinicians were considered to ensure real-world guidance. New medications may be introduced, while established medications may be withdrawn, and the evidence base for their administration requirements is changing. Line material is also evolving. For example, thermoplastic polyurethane is expected to replace polyvinyl chloride (manufactured without di(2-ethylhexyl) phthalate). No study can be exhaustive, but a review period of 24 months was considered by the panel to be sufficient to cover these changes in the environment. It is also difficult to cover, in any study or review, all regions and countries. There was no panel member from North America, but there was a strong reliance on evidence presented to the Food and Drug Administration.

There was no consensus for just over one-third of the medications assessed. Therefore, the global nature of the panel highlighted a diversity of opinion and reflects the issues faced by compounding pharmacists and nursing staff. These unresolved medications can be prioritized in the planning of the next Delphi panel (ie, at the 24-month review period). The potential for machine learning to assist in synthesizing evidence dossiers for these medications could include incorporating evidence from languages other than English. A wider catchment of generic manufacturers’ package inserts and extracts from industry publications could also aid in the creation of consensus for these medications. Our general advice for these medications is to follow a conservative approach for light protection during storage, and to consider the duration of infusion when determining whether light protection is needed during administration. For filtration, there is emerging evidence that higher-concentration infusions used at lower rates [23], and the use of 1-lumen for multiple infusions may contribute to vascular access occlusion, and that the use of 0.2-μm filters may reduce this risk [24,25]. Reviewing medications at high and low concentrations, and the effect of concentration on the need for specific line materials, filtration, and light protection, could be included in subsequent reviews.

The dossier of evidence was prepared by JW and AD. Given the extent and variety of the evidence reviewed, it is inevitable that omissions were made. This was evident in the panelists’ recommendations to incorporate new medications or remove those that were no longer actively marketed. In the future, these dossiers should be coproduced by a broader panel of experts, independent of the Delphi panel.

A further important limitation lies in the Delphi process. Delphi exercises are labor-intensive, and there is evidence that cycles of consensus reach diminishing returns once they extend beyond 3 cycles [26,27]. There is also the potential for bias within the panel; however, its members are selected, and it is impossible to ensure that the outcome would be the same if panel membership were different. Therefore, we must reiterate that this tool is not a universal consensus but is representative of the views of the 8 subject matter experts who took part.

Generic manufacturers are allowed—and often do use—different inactive excipients in their formulations. Excipients can influence properties, such as solubility, pH, particulate formation, adsorption to plastic, or light sensitivity. Therefore, 2 generics labeled as “the same drug” may behave differently. The ultimate goal would be to consult each generic’s prescribing information or stability study, as uniform performance across all brands cannot be expected. In this work, we had to narrow down the scope of validity to the sources we have used in our dossier.

### Recommendations for Future Research

While we have produced an outline framework for decision support for these medications, it has not been tested for utility in real-world settings. Therefore, we recommend a program of study evaluating the utility of this tool in health care practice environments. There are also myriad ways in which this can be deployed, either as simple lookup tables in low-resource settings or through integration into electronic patient records and prescribing systems.

### Conclusions

We feel that our approach of restricting ourselves to the fundamental information required by bedside clinicians was vindicated, as casting the net wider to include other parameters, such as concentration and solvent, would arguably overcomplicate the output we were seeking from the study and any tools derived from it. Our experience here matched that of other authors who found that the “temptation to be comprehensive by including every theoretically possible scenario may be counterproductive. Also, being selective allows larger numbers of cues to be considered explicitly, which should be an advantage in terms of reliability” [17]. As noted previously under the “working assumptions” in the Methods section, panel members were instructed to use the most conservative scenarios for each medication being assessed; for example, higher-concentration solutions would be assessed as part of the requirements for filtration, administration line material, and light protection.

One immediate step that could use the results of the consensus study would be to label sterile compounded products with information for the end user, such as “protect from light during storage and administration.”

The output of the study is also being used to underpin algorithms for a Python-based prototype bedside app through which clinicians can identify the correct administration line characteristics required for both primary and secondary administration after entering the medication name into the app. Such a tool could also help address the perennial problems of clinicians spending considerable amounts of time looking for information and guidance or addressing knowledge and information deficits with recalled facts gathered from past experience, including informal and formal teaching, interactions with colleagues, and the traditions of care within the unit where they work [28]. This was one reason we prioritized simplicity in our project plan and method, as we aimed to ensure that the results could be simply disseminated to the point of care. Given the 2022 study’s findings that knowledge of administration line material type and characteristic requirements (light protection and filtration), even among specialist nurses, was poor [1]; the immense (and well-established) challenges associated with cascading information through organizations [29]; and the fact that end users would be predominantly nurses (the largest component of the tertiary health care workforce), such a point-of-care line selection tool could be very valuable [30].

**Figure 1:**
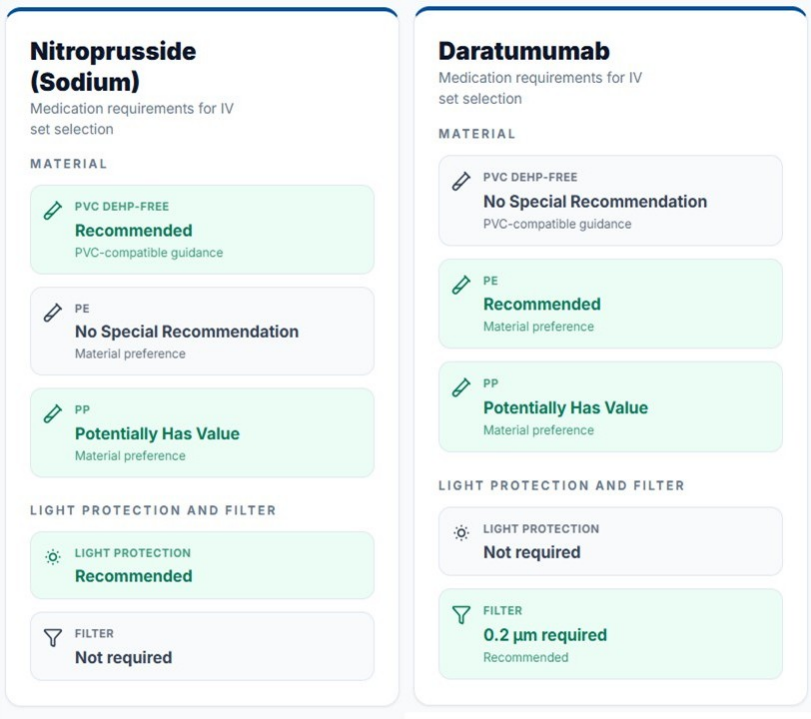
Screenshots of a prototype app to be used bedside for selecting administration line characteristics.

We hope to fully evaluate this bedside tool in a study within the next year. In its current unreleased form, a medication can be selected on a smartphone app from a drop-down list of the medications on which consensus was secured, and the required properties of the line in terms of material, light protection, and filtration are displayed (Figure 1). This will need to be extended to the inventory of administration lines and IV infusion pumps available to the end user to direct them to the most appropriate options for the administration of the medication.

## Appendix

Multimedia Appendix 1 Sample evidence dossier.

Multimedia Appendix 2 Consensus recommendations after a 3-round Delphi study.

## Abbreviations

IV: intravenous
