# Supplementary material for: Good Clinical Practice Guidance for Line Materials, Filtration, and Light Protection in Intravenous Medication Administration: Modified Delphi Consensus Study
Source: JMIR Hum Factors. 2026 Jun 10;13:e88333. doi: 10.2196/88333 (PMC13294651; doi:10.2196/88333)
Supplement: Multimedia Appendix 1 [file humanfactors_v13i1e88333_app1.docx]

**Appendix One: Sample Evidence Dossier.**
