# Supplementary material for: Good Clinical Practice Guidance for Line Materials, Filtration, and Light Protection in Intravenous Medication Administration: Modified Delphi Consensus Study
Source: JMIR Hum Factors. 2026 Jun 10;13:e88333. doi: 10.2196/88333 (PMC13294651; doi:10.2196/88333)
Supplement: Multimedia Appendix 2 [file humanfactors_v13i1e88333_app2.docx]

**Appendix Two: Recommendations Consensus after Three Rounds.**

| **PVC DEHP Free** | | | | |
| --- | --- | --- | --- | --- |
| **Medication** | **Material Statement** | **SD** | **Score Align** | **Consensus Opinion** |
| Fotemustine | Yes | 0.000 | 100.0% | Recommended |
| Siltuximab | Yes | 0.000 | 100.0% | Recommended |
| Busulfan | Yes | 0.000 | 100.0% | Recommended |
| Cisplatin | Yes | 0.000 | 100.0% | Recommended |
| Erlotynib | Yes | 0.000 | 100.0% | Recommended |
| Gemtuzumab ozogamicin | Yes | 0.000 | 100.0% | Recommended |
| Inotuzumab ozogamici | Yes | 0.000 | 100.0% | Recommended |
| Isatuximab | Yes | 0.000 | 100.0% | Recommended |
| Leucovorin (calcium folinate) | Yes | 0.000 | 100.0% | Recommended |
| Lurbinectedin | Yes | 0.000 | 100.0% | Recommended |
| Nelarabine | Yes | 0.000 | 100.0% | Recommended |
| Obinutuzumab | Yes | 0.000 | 100.0% | Recommended |
| Paclitaxel Albumin bound | Yes | 0.000 | 100.0% | Recommended |
| Ranitidine | Yes | 0.000 | 100.0% | Recommended |
| Trabectedin | Yes | 0.000 | 100.0% | Recommended |
| Carmustine | Yes | 0.373 | 81.4% | Recommended |
| Acetylcysteine | Yes | 0.373 | 81.4% | Recommended |
| Adrenaline (Epinephrine) | Yes | 0.373 | 81.4% | Recommended |
| Aldesleukin (Interleukin-2) | Yes | 0.373 | 81.4% | Recommended |
| Alemtuzumab | Yes | 0.373 | 81.4% | Recommended |
| Alteplase | Yes | 0.373 | 81.4% | Recommended |
| Asparaginase Erwinia | Yes | 0.373 | 81.4% | Recommended |
| Atezolizumab | Yes | 0.373 | 81.4% | Recommended |
| Avelumab | Yes | 0.373 | 81.4% | Recommended |
| Blinatumomab | Yes | 0.373 | 81.4% | Recommended |
| Cetuximab | Yes | 0.373 | 81.4% | Recommended |
| Cladribine | Yes | 0.373 | 81.4% | Recommended |
| Copanlisib | Yes | 0.373 | 81.4% | Recommended |
| Cyclophosphamide | Yes | 0.373 | 81.4% | Recommended |
| Cytarabine | Yes | 0.373 | 81.4% | Recommended |
| Dacarbazine | Yes | 0.373 | 81.4% | Recommended |
| Dactinomycin | Yes | 0.373 | 81.4% | Recommended |
| Denileukin Diftitox | Yes | 0.373 | 81.4% | Recommended |
| Dexamethasone | Yes | 0.373 | 81.4% | Recommended |
| Dinutuximab | Yes | 0.373 | 81.4% | Recommended |
| DOPamine Hydrochloride | Yes | 0.373 | 81.4% | Recommended |
| Doxorubicin | Yes | 0.373 | 81.4% | Recommended |
| Doxorubicin liposomal | Yes | 0.373 | 81.4% | Recommended |
| Durvalumab | Yes | 0.373 | 81.4% | Recommended |
| Elotuzumab | Yes | 0.373 | 81.4% | Recommended |
| Enfortumab vedotin-ejfv | Yes | 0.373 | 81.4% | Recommended |
| Epirubicin | Yes | 0.373 | 81.4% | Recommended |
| Eribulin | Yes | 0.373 | 81.4% | Recommended |
| Esmolol Hydrochloride | Yes | 0.373 | 81.4% | Recommended |
| Etoposide phosphate | Yes | 0.373 | 81.4% | Recommended |
| Fentanyl Citrate | Yes | 0.373 | 81.4% | Recommended |
| Filgrastim | Yes | 0.373 | 81.4% | Recommended |
| Fludarabine | Yes | 0.373 | 81.4% | Recommended |
| Fluorouracil (5-FU) | Yes | 0.373 | 81.4% | Recommended |
| Folic Acid | Yes | 0.373 | 81.4% | Recommended |
| Furosemide | Yes | 0.373 | 81.4% | Recommended |
| Gemcitabine | Yes | 0.373 | 81.4% | Recommended |
| Gentamicin Sulfate | Yes | 0.373 | 81.4% | Recommended |
| Heparin Sodium | Yes | 0.373 | 81.4% | Recommended |
| Ifosfamide | Yes | 0.373 | 81.4% | Recommended |
| Infliximab | Yes | 0.373 | 81.4% | Recommended |
| Ipilimumab | Yes | 0.373 | 81.4% | Recommended |
| Irinotecan hydrchloride | Yes | 0.373 | 81.4% | Recommended |
| Irinotecan liposomal | Yes | 0.373 | 81.4% | Recommended |
| Labetalol Hydrochloride | Yes | 0.373 | 81.4% | Recommended |
| Lenograstim | Yes | 0.373 | 81.4% | Recommended |
| Levofloxacin | Yes | 0.373 | 81.4% | Recommended |
| Magnesium sulphate | Yes | 0.373 | 81.4% | Recommended |
| Margetuximab-cmkb | Yes | 0.373 | 81.4% | Recommended |
| Mechlorethamine | Yes | 0.373 | 81.4% | Recommended |
| Melphalan | Yes | 0.373 | 81.4% | Recommended |
| Meropenem | Yes | 0.373 | 81.4% | Recommended |
| Mesna | Yes | 0.373 | 81.4% | Recommended |
| Methotrexate | Yes | 0.373 | 81.4% | Recommended |
| Methylprednisolone sodium succinate | Yes | 0.373 | 81.4% | Recommended |
| Midazolam Hydrochloride | Yes | 0.373 | 81.4% | Recommended |
| Milrinone Lactate | Yes | 0.373 | 81.4% | Recommended |
| Mitoxantrone | Yes | 0.373 | 81.4% | Recommended |
| Mogamulizumab | Yes | 0.373 | 81.4% | Recommended |
| Morphine Hydrochloride | Yes | 0.373 | 81.4% | Recommended |
| Naxitamab-gqgk | Yes | 0.373 | 81.4% | Recommended |
| Nitroprusside (Sodium) | Yes | 0.373 | 81.4% | Recommended |
| Nivolumab | Yes | 0.373 | 81.4% | Recommended |
| Noradrenaline Acid Tartrate / Norepinephrine | Yes | 0.373 | 81.4% | Recommended |
| Omeprazole Sodium | Yes | 0.373 | 81.4% | Recommended |
| Ondansetron Hydrochloride | Yes | 0.373 | 81.4% | Recommended |
| Oxaliplatin | Yes | 0.373 | 81.4% | Recommended |
| Pamidronate | Yes | 0.373 | 81.4% | Recommended |
| Panitumumab | Yes | 0.373 | 81.4% | Recommended |
| Paracetamol | Yes | 0.373 | 81.4% | Recommended |
| Pegaspargase | Yes | 0.373 | 81.4% | Recommended |
| Pemetrexed | Yes | 0.373 | 81.4% | Recommended |
| Pentostatin | Yes | 0.373 | 81.4% | Recommended |
| Pertuzumab | Yes | 0.373 | 81.4% | Recommended |
| Piperacillin Sodium-Tazobactam Sodium | Yes | 0.373 | 81.4% | Recommended |
| Polatuzumab | Yes | 0.373 | 81.4% | Recommended |
| Potassium Chloride | Yes | 0.373 | 81.4% | Recommended |
| Procainamide Hydrochloride | Yes | 0.373 | 81.4% | Recommended |
| Propofol | Yes | 0.373 | 81.4% | Recommended |
| Ramucirumab | Yes | 0.373 | 81.4% | Recommended |
| Rifampicin | Yes | 0.373 | 81.4% | Recommended |
| Rituximab | Yes | 0.373 | 81.4% | Recommended |
| Rocuronium Bromide | Yes | 0.373 | 81.4% | Recommended |
| Romidepsin | Yes | 0.373 | 81.4% | Recommended |
| Sacituzumab Govitecan | Yes | 0.373 | 81.4% | Recommended |
| Teicoplanin | Yes | 0.373 | 81.4% | Recommended |
| Thiotepa | Yes | 0.373 | 81.4% | Recommended |
| Tocilizumab | Yes | 0.373 | 81.4% | Recommended |
| Topotecan | Yes | 0.373 | 81.4% | Recommended |
| Trastuzumab | Yes | 0.373 | 81.4% | Recommended |
| Trastuzumab emtansine | Yes | 0.373 | 81.4% | Recommended |
| Treosulfan | Yes | 0.373 | 81.4% | Recommended |
| Vancomycin Hydrochloride | Yes | 0.373 | 81.4% | Recommended |
| Vecuronium Bromide | Yes | 0.373 | 81.4% | Recommended |
| Vinblastine | Yes | 0.373 | 81.4% | Recommended |
| Vincristine | Yes | 0.373 | 81.4% | Recommended |
| Vindesine | Yes | 0.373 | 81.4% | Recommended |
| Vinflunine | Yes | 0.373 | 81.4% | Recommended |
| Zoledronic Acid | Yes | 0.373 | 81.4% | Recommended |
| Interferon Alfa2b | Yes | 0.400 | 80.0% | Recommended |
| Vinorelbine | Yes | 0.400 | 80.0% | Recommended |
| Daunorubicin- Cytarabine liposomal | Yes | 0.000 | 100.0% | Recommended |
| Aflibercept | Yes | 0.373 | 81.4% | Recommended |
| Bevacizumab | Yes | 0.373 | 81.4% | Recommended |
| Bleomycin | Yes | 0.373 | 81.4% | Recommended |
| Brentuximab Vedotin | Yes | 0.373 | 81.4% | Recommended |
| Calcium folinate | Yes | 0.373 | 81.4% | Recommended |
| Carboplatin | Yes | 0.373 | 81.4% | Recommended |
| Daunorubicin | Yes | 0.373 | 81.4% | Recommended |
| Daunorubicin liposomal | Yes | 0.373 | 81.4% | Recommended |
| DOBUTamine Hydrochloride | Yes | 0.373 | 81.4% | Recommended |
| Idarubicin | Yes | 0.373 | 81.4% | Recommended |
| Mitomycin | Yes | 0.373 | 81.4% | Recommended |
| Ofatumumab | Yes | 0.373 | 81.4% | Recommended |
| Paclitaxel | Yes | 0.373 | 81.4% | Recommended |
| Salbutamol Sulfate | Yes | 0.373 | 81.4% | Recommended |
| Temsirolimus | Yes | 0.373 | 81.4% | Recommended |
| Bendamustine | Yes | 0.471 | 76.4% | No Specific Recommendation |
| Amiodarone Hydrochloride | Yes | 0.745 | 62.7% | No Specific Recommendation |
| Azacitidine | Yes | 0.745 | 62.7% | No Specific Recommendation |
| Cimetidine | Yes | 0.745 | 62.7% | No Specific Recommendation |
| Digoxin | Yes | 0.745 | 62.7% | No Specific Recommendation |
| Diphenhydramine | Yes | 0.745 | 62.7% | No Specific Recommendation |
| Disodium folinate | Yes | 0.745 | 62.7% | No Specific Recommendation |
| Etoposide | Yes | 0.745 | 62.7% | No Specific Recommendation |
| Remifentanil Hydrochloride | Yes | 0.745 | 62.7% | No Specific Recommendation |
| Arsenic Trioxide | Yes | 0.764 | 61.8% | No Specific Recommendation |
| Belinostat | Yes | 0.764 | 61.8% | No Specific Recommendation |
| Bicarbonate Sodium (Sodium Bicarbonate) | Yes | 0.764 | 61.8% | No Specific Recommendation |
| Bortezomib | Yes | 0.764 | 61.8% | No Specific Recommendation |
| Carfilzomib | Yes | 0.764 | 61.8% | No Specific Recommendation |
| Cemiplimab | Yes | 0.764 | 61.8% | No Specific Recommendation |
| Chlorphenamine | Yes | 0.764 | 61.8% | No Specific Recommendation |
| Clofarabine | Yes | 0.764 | 61.8% | No Specific Recommendation |
| Decitabine | Yes | 0.764 | 61.8% | No Specific Recommendation |
| Insulin Regular | Yes | 0.764 | 61.8% | No Specific Recommendation |
| Necitumumab | Yes | 0.764 | 61.8% | No Specific Recommendation |
| Pembrolizumab | Yes | 0.764 | 61.8% | No Specific Recommendation |
| Pralatrexate | Yes | 0.764 | 61.8% | No Specific Recommendation |
| Raltitrexed | Yes | 0.764 | 61.8% | No Specific Recommendation |
| Streptozocin | Yes | 0.764 | 61.8% | No Specific Recommendation |
| Tagraxofusp | Yes | 0.764 | 61.8% | No Specific Recommendation |
| Asparaginase (E. coli) | Yes | 0.898 | 55.1% | No Specific Recommendation |
| L-asparaginase | Yes | 0.898 | 55.1% | No Specific Recommendation |
| Interferon alfa-2a | Yes | 0.943 | 52.9% | No Specific Recommendation |
| Amsacrine | Yes | 1.106 | 44.7% | No Specific Recommendation |
| Trastuzumab deruxtecan | Yes | 1.118 | 44.1% | No Specific Recommendation |
| Ibritumomab | Yes | 1.462 | 26.9% | No Specific Recommendation |
| Lutetium (177Lu) Oxodotreotide | Yes | 1.462 | 26.9% | No Specific Recommendation |
| Mannitol | Yes | 1.462 | 26.9% | No Specific Recommendation |
| Radium 223 dichloride | Yes | 1.462 | 26.9% | No Specific Recommendation |
| Belantamab mafodotin | Yes | 1.491 | 25.5% | No Specific Recommendation |
| Daratumumab | Yes | 1.491 | 25.5% | No Specific Recommendation |
| Docetaxel | No | 1.491 | 25.5% | No Specific Recommendation |
| Nitroglycerin | Yes | 1.491 | 25.5% | No Specific Recommendation |
| Tafasitamab-cxix | Yes | 1.491 | 25.5% | No Specific Recommendation |
| Diazepam | Yes | 1.599 | 20.1% | No Specific Recommendation |
| Cabazitaxel | Yes | 1.886 | 5.7% | No Specific Recommendation |
| Nimodipine | Yes | 1.886 | 5.7% | No Specific Recommendation |

| **Polyethylene Administration Line Material** | | | | |
| --- | --- | --- | --- | --- |
| **Medication** | **Material Statement** | **SD** | **Score Align** | **Consensus Opinion** |
| Alemtuzumab | Yes | 0.000 | 100.0% | Recommended |
| Arsenic Trioxide | Yes | 0.000 | 100.0% | Recommended |
| Asparaginase (E. coli) | Yes | 0.000 | 100.0% | Recommended |
| Atezolizumab | Yes | 0.000 | 100.0% | Recommended |
| Avelumab | Yes | 0.000 | 100.0% | Recommended |
| Bicarbonate Sodium (Sodium Bicarbonate) | Yes | 0.000 | 100.0% | Recommended |
| Busulfan | Yes | 0.000 | 100.0% | Recommended |
| Cabazitaxel | Yes | 0.000 | 100.0% | Recommended |
| Carfilzomib | Yes | 0.000 | 100.0% | Recommended |
| Cetuximab | Yes | 0.000 | 100.0% | Recommended |
| Cisplatin | Yes | 0.000 | 100.0% | Recommended |
| Cladribine | Yes | 0.000 | 100.0% | Recommended |
| Clofarabine | Yes | 0.000 | 100.0% | Recommended |
| Cyclophosphamide | Yes | 0.000 | 100.0% | Recommended |
| Cytarabine | Yes | 0.000 | 100.0% | Recommended |
| Dacarbazine | Yes | 0.000 | 100.0% | Recommended |
| Daratumumab | Yes | 0.000 | 100.0% | Recommended |
| Decitabine | Yes | 0.000 | 100.0% | Recommended |
| Denileukin Diftitox | Yes | 0.000 | 100.0% | Recommended |
| Dexamethasone | Yes | 0.000 | 100.0% | Recommended |
| Dinutuximab | Yes | 0.000 | 100.0% | Recommended |
| DOPamine Hydrochloride | Yes | 0.000 | 100.0% | Recommended |
| Doxorubicin | Yes | 0.000 | 100.0% | Recommended |
| Durvalumab | Yes | 0.000 | 100.0% | Recommended |
| Elotuzumab | Yes | 0.000 | 100.0% | Recommended |
| Enfortumab vedotin-ejfv | Yes | 0.000 | 100.0% | Recommended |
| Epirubicin | Yes | 0.000 | 100.0% | Recommended |
| Eribulin | Yes | 0.000 | 100.0% | Recommended |
| Erlotynib | Yes | 0.000 | 100.0% | Recommended |
| Esmolol Hydrochloride | Yes | 0.000 | 100.0% | Recommended |
| Fentanyl Citrate | Yes | 0.000 | 100.0% | Recommended |
| Filgrastim | Yes | 0.000 | 100.0% | Recommended |
| Fludarabine | Yes | 0.000 | 100.0% | Recommended |
| Fluorouracil (5-FU) | Yes | 0.000 | 100.0% | Recommended |
| Folic Acid | Yes | 0.000 | 100.0% | Recommended |
| Furosemide | Yes | 0.000 | 100.0% | Recommended |
| Gemcitabine | Yes | 0.000 | 100.0% | Recommended |
| Gemtuzumab ozogamicin | Yes | 0.000 | 100.0% | Recommended |
| Gentamicin Sulfate | Yes | 0.000 | 100.0% | Recommended |
| Heparin Sodium | Yes | 0.000 | 100.0% | Recommended |
| Idarubicin | Yes | 0.000 | 100.0% | Recommended |
| Ifosfamide | Yes | 0.000 | 100.0% | Recommended |
| Inotuzumab ozogamici | Yes | 0.000 | 100.0% | Recommended |
| Ipilimumab | Yes | 0.000 | 100.0% | Recommended |
| Irinotecan hydrchloride | Yes | 0.000 | 100.0% | Recommended |
| Isatuximab | Yes | 0.000 | 100.0% | Recommended |
| Labetalol Hydrochloride | Yes | 0.000 | 100.0% | Recommended |
| Leucovorin (calcium folinate) | Yes | 0.000 | 100.0% | Recommended |
| Levofloxacin | Yes | 0.000 | 100.0% | Recommended |
| Lurbinectedin | Yes | 0.000 | 100.0% | Recommended |
| Magnesium sulphate | Yes | 0.000 | 100.0% | Recommended |
| Mannitol | Yes | 0.000 | 100.0% | Recommended |
| Margetuximab-cmkb | Yes | 0.000 | 100.0% | Recommended |
| Melphalan | Yes | 0.000 | 100.0% | Recommended |
| Meropenem | Yes | 0.000 | 100.0% | Recommended |
| Mesna | Yes | 0.000 | 100.0% | Recommended |
| Methotrexate | Yes | 0.000 | 100.0% | Recommended |
| Methylprednisolone sodium succinate | Yes | 0.000 | 100.0% | Recommended |
| Midazolam Hydrochloride | Yes | 0.000 | 100.0% | Recommended |
| Milrinone Lactate | Yes | 0.000 | 100.0% | Recommended |
| Mitoxantrone | Yes | 0.000 | 100.0% | Recommended |
| Mogamulizumab | Yes | 0.000 | 100.0% | Recommended |
| Morphine Hydrochloride | Yes | 0.000 | 100.0% | Recommended |
| Nimodipine | Yes | 0.000 | 100.0% | Recommended |
| Nivolumab | Yes | 0.000 | 100.0% | Recommended |
| Noradrenaline Acid Tartrate / Norepinephrine | Yes | 0.000 | 100.0% | Recommended |
| Obinutuzumab | Yes | 0.000 | 100.0% | Recommended |
| Omeprazole Sodium | Yes | 0.000 | 100.0% | Recommended |
| Ondansetron Hydrochloride | Yes | 0.000 | 100.0% | Recommended |
| Oxaliplatin | Yes | 0.000 | 100.0% | Recommended |
| Paclitaxel | Yes | 0.000 | 100.0% | Recommended |
| Paclitaxel Albumin bound | Yes | 0.000 | 100.0% | Recommended |
| Panitumumab | Yes | 0.000 | 100.0% | Recommended |
| Paracetamol | Yes | 0.000 | 100.0% | Recommended |
| Pegaspargase | Yes | 0.000 | 100.0% | Recommended |
| Pembrolizumab | Yes | 0.000 | 100.0% | Recommended |
| Pemetrexed | Yes | 0.000 | 100.0% | Recommended |
| Pertuzumab | Yes | 0.000 | 100.0% | Recommended |
| Piperacillin Sodium-Tazobactam Sodium | Yes | 0.000 | 100.0% | Recommended |
| Polatuzumab | Yes | 0.000 | 100.0% | Recommended |
| Potassium Chloride | Yes | 0.000 | 100.0% | Recommended |
| Propofol | Yes | 0.000 | 100.0% | Recommended |
| Ramucirumab | Yes | 0.000 | 100.0% | Recommended |
| Ranitidine | Yes | 0.000 | 100.0% | Recommended |
| Rituximab | Yes | 0.000 | 100.0% | Recommended |
| Rocuronium Bromide | Yes | 0.000 | 100.0% | Recommended |
| Romidepsin | Yes | 0.000 | 100.0% | Recommended |
| Sacituzumab Govitecan | Yes | 0.000 | 100.0% | Recommended |
| Streptozocin | Yes | 0.000 | 100.0% | Recommended |
| Tafasitamab-cxix | Yes | 0.000 | 100.0% | Recommended |
| Temsirolimus | Yes | 0.000 | 100.0% | Recommended |
| Thiotepa | Yes | 0.000 | 100.0% | Recommended |
| Tocilizumab | Yes | 0.000 | 100.0% | Recommended |
| Topotecan | Yes | 0.000 | 100.0% | Recommended |
| Trabectedin | Yes | 0.000 | 100.0% | Recommended |
| Trastuzumab | Yes | 0.000 | 100.0% | Recommended |
| Trastuzumab emtansine | Yes | 0.000 | 100.0% | Recommended |
| Treosulfan | Yes | 0.000 | 100.0% | Recommended |
| Vancomycin Hydrochloride | Yes | 0.000 | 100.0% | Recommended |
| Vecuronium Bromide | Yes | 0.000 | 100.0% | Recommended |
| Vinblastine | Yes | 0.000 | 100.0% | Recommended |
| Vincristine | Yes | 0.000 | 100.0% | Recommended |
| Vinflunine | Yes | 0.000 | 100.0% | Recommended |
| Vinorelbine | Yes | 0.000 | 100.0% | Recommended |
| Zoledronic Acid | Yes | 0.000 | 100.0% | Recommended |
| Calcium folinate | Yes | 0.373 | 81.4% | Recommended |
| Carboplatin | Yes | 0.373 | 81.4% | Recommended |
| Carmustine | Yes | 0.373 | 81.4% | Recommended |
| Daunorubicin | Yes | 0.373 | 81.4% | Recommended |
| Diazepam | Yes | 0.373 | 81.4% | Recommended |
| DOBUTamine Hydrochloride | Yes | 0.373 | 81.4% | Recommended |
| Insulin Regular | Yes | 0.373 | 81.4% | Recommended |
| Mitomycin | Yes | 0.373 | 81.4% | Recommended |
| Nitroglycerin | Yes | 0.373 | 81.4% | Recommended |
| Amiodarone Hydrochloride | Yes | 0.373 | 81.4% | Recommended |
| Blinatumomab | Yes | 0.373 | 81.4% | Recommended |
| Etoposide phosphate | Yes | 0.373 | 81.4% | Recommended |
| Bendamustine | Yes | 0.000 | 100.0% | Recommended |
| Bevacizumab | Yes | 0.000 | 100.0% | Recommended |
| Bleomycin | Yes | 0.000 | 100.0% | Recommended |
| Infliximab | Yes | 0.000 | 100.0% | Recommended |
| L-asparaginase | Yes | 0.000 | 100.0% | Recommended |
| Ofatumumab | Yes | 0.373 | 81.4% | Recommended |
| Asparaginase Erwinia | Yes | 0.745 | 62.7% | No Specific Recommendation |
| Brentuximab Vedotin | Yes | 0.745 | 62.7% | No Specific Recommendation |
| Dactinomycin | Yes | 0.745 | 62.7% | No Specific Recommendation |
| Daunorubicin liposomal | Yes | 0.745 | 62.7% | No Specific Recommendation |
| Doxorubicin liposomal | Yes | 0.745 | 62.7% | No Specific Recommendation |
| Irinotecan liposomal | Yes | 0.745 | 62.7% | No Specific Recommendation |
| Lenograstim | Yes | 0.745 | 62.7% | No Specific Recommendation |
| Pamidronate | Yes | 0.745 | 62.7% | No Specific Recommendation |
| Salbutamol Sulfate | Yes | 0.745 | 62.7% | No Specific Recommendation |
| Teicoplanin | Yes | 0.745 | 62.7% | No Specific Recommendation |
| Aldesleukin (Interleukin-2) | Yes | 0.764 | 61.8% | No Specific Recommendation |
| Daunorubicin- Cytarabine liposomal | Yes | 0.764 | 61.8% | No Specific Recommendation |
| Fotemustine | Yes | 1.528 | 23.6% | No Specific Recommendation |
| Siltuximab | Yes | 1.528 | 23.6% | No Specific Recommendation |
| Chlorphenamine | Yes | 0.898 | 55.1% | No Specific Recommendation |
| Acetylcysteine | Yes | 0.943 | 52.9% | No Specific Recommendation |
| Aflibercept | Yes | 0.943 | 52.9% | No Specific Recommendation |
| Alteplase | Yes | 0.943 | 52.9% | No Specific Recommendation |
| Amsacrine | Yes | 0.943 | 52.9% | No Specific Recommendation |
| Belinostat | Yes | 0.943 | 52.9% | No Specific Recommendation |
| Bortezomib | Yes | 0.943 | 52.9% | No Specific Recommendation |
| Cemiplimab | Yes | 0.943 | 52.9% | No Specific Recommendation |
| Copanlisib | Yes | 0.943 | 52.9% | No Specific Recommendation |
| Disodium folinate | Yes | 0.943 | 52.9% | No Specific Recommendation |
| Ibritumomab | Yes | 0.943 | 52.9% | No Specific Recommendation |
| Mechlorethamine | Yes | 0.943 | 52.9% | No Specific Recommendation |
| Naxitamab-gqgk | Yes | 0.943 | 52.9% | No Specific Recommendation |
| Necitumumab | Yes | 0.943 | 52.9% | No Specific Recommendation |
| Nelarabine | Yes | 0.943 | 52.9% | No Specific Recommendation |
| Nitroprusside (Sodium) | Yes | 0.943 | 52.9% | No Specific Recommendation |
| Pentostatin | Yes | 0.943 | 52.9% | No Specific Recommendation |
| Pralatrexate | Yes | 0.943 | 52.9% | No Specific Recommendation |
| Procainamide Hydrochloride | Yes | 0.943 | 52.9% | No Specific Recommendation |
| Raltitrexed | Yes | 0.943 | 52.9% | No Specific Recommendation |
| Rifampicin | Yes | 0.943 | 52.9% | No Specific Recommendation |
| Tagraxofusp | Yes | 0.943 | 52.9% | No Specific Recommendation |
| Vindesine | Yes | 0.943 | 52.9% | No Specific Recommendation |
| Cimetidine | Yes | 1.000 | 50.0% | No Specific Recommendation |
| Digoxin | Yes | 1.000 | 50.0% | No Specific Recommendation |
| Diphenhydramine | Yes | 1.000 | 50.0% | No Specific Recommendation |
| Remifentanil Hydrochloride | Yes | 1.000 | 50.0% | No Specific Recommendation |
| Azacitidine | Yes | 1.213 | 39.3% | No Specific Recommendation |
| Belantamab mafodotin | Yes | 1.213 | 39.3% | No Specific Recommendation |
| Trastuzumab deruxtecan | Yes | 1.213 | 39.3% | No Specific Recommendation |
| Docetaxel | Yes | 1.491 | 25.5% | No Specific Recommendation |
| Etoposide | Yes | 1.491 | 25.5% | No Specific Recommendation |
| Interferon alfa-2a | Yes | 1.491 | 25.5% | No Specific Recommendation |
| Adrenaline (Epinephrine) | Yes | 1.528 | 23.6% | No Specific Recommendation |
| Interferon Alfa2b | Yes | 1.528 | 23.6% | No Specific Recommendation |
| Lutetium (177Lu) Oxodotreotide | Yes | 1.528 | 23.6% | No Specific Recommendation |
| Radium 223 dichloride | Yes | 1.528 | 23.6% | No Specific Recommendation |

| **Polypropylene Administration Line Material** | | | | |
| --- | --- | --- | --- | --- |
| **Medication** | **Material Statement** | **SD** | **Score Align** | **Consensus Opinion** |
| Atezolizumab | Yes | 0.000 | 100.0% | Recommended |
| Avelumab | Yes | 0.000 | 100.0% | Recommended |
| Busulfan | Yes | 0.000 | 100.0% | Recommended |
| Carfilzomib | Yes | 0.000 | 100.0% | Recommended |
| Cetuximab | Yes | 0.000 | 100.0% | Recommended |
| Cyclophosphamide | Yes | 0.000 | 100.0% | Recommended |
| Decitabine | Yes | 0.000 | 100.0% | Recommended |
| Denileukin Diftitox | Yes | 0.000 | 100.0% | Recommended |
| Dexamethasone | Yes | 0.000 | 100.0% | Recommended |
| Elotuzumab | Yes | 0.000 | 100.0% | Recommended |
| Fludarabine | Yes | 0.000 | 100.0% | Recommended |
| Fluorouracil (5-FU) | Yes | 0.000 | 100.0% | Recommended |
| Ifosfamide | Yes | 0.000 | 100.0% | Recommended |
| Irinotecan hydrchloride | Yes | 0.000 | 100.0% | Recommended |
| L-asparaginase | Yes | 0.000 | 100.0% | Recommended |
| Lurbinectedin | Yes | 0.000 | 100.0% | Recommended |
| Margetuximab-cmkb | Yes | 0.000 | 100.0% | Recommended |
| Meropenem | Yes | 0.000 | 100.0% | Recommended |
| Mesna | Yes | 0.000 | 100.0% | Recommended |
| Methotrexate | Yes | 0.000 | 100.0% | Recommended |
| Methylprednisolone sodium succinate | Yes | 0.000 | 100.0% | Recommended |
| Mitomycin | Yes | 0.000 | 100.0% | Recommended |
| Noradrenaline Acid Tartrate / Norepinephrine | Yes | 0.000 | 100.0% | Recommended |
| Obinutuzumab | Yes | 0.000 | 100.0% | Recommended |
| Omeprazole Sodium | Yes | 0.000 | 100.0% | Recommended |
| Ondansetron Hydrochloride | Yes | 0.000 | 100.0% | Recommended |
| Paclitaxel Albumin bound | Yes | 0.000 | 100.0% | Recommended |
| Paracetamol | Yes | 0.000 | 100.0% | Recommended |
| Pembrolizumab | Yes | 0.000 | 100.0% | Recommended |
| Pemetrexed | Yes | 0.000 | 100.0% | Recommended |
| Pertuzumab | Yes | 0.000 | 100.0% | Recommended |
| Piperacillin Sodium-Tazobactam Sodium | Yes | 0.000 | 100.0% | Recommended |
| Potassium Chloride | Yes | 0.000 | 100.0% | Recommended |
| Ranitidine | Yes | 0.000 | 100.0% | Recommended |
| Salbutamol Sulfate | Yes | 0.000 | 100.0% | Recommended |
| Temsirolimus | Yes | 0.000 | 100.0% | Recommended |
| Thiotepa | Yes | 0.000 | 100.0% | Recommended |
| Tocilizumab | Yes | 0.000 | 100.0% | Recommended |
| Topotecan | Yes | 0.000 | 100.0% | Recommended |
| Trabectedin | Yes | 0.000 | 100.0% | Recommended |
| Trastuzumab emtansine | Yes | 0.000 | 100.0% | Recommended |
| Vancomycin Hydrochloride | Yes | 0.000 | 100.0% | Recommended |
| Vecuronium Bromide | Yes | 0.000 | 100.0% | Recommended |
| Vinblastine | Yes | 0.000 | 100.0% | Recommended |
| Vincristine | Yes | 0.000 | 100.0% | Recommended |
| Vindesine | Yes | 0.000 | 100.0% | Recommended |
| Vinflunine | Yes | 0.000 | 100.0% | Recommended |
| Acetylcysteine | Yes | 0.373 | 81.4% | Recommended |
| Asparaginase (E. coli) | Yes | 0.373 | 81.4% | Recommended |
| Clofarabine | Yes | 0.373 | 81.4% | Recommended |
| Epirubicin | Yes | 0.373 | 81.4% | Recommended |
| Morphine Hydrochloride | Yes | 0.373 | 81.4% | Recommended |
| Pegaspargase | Yes | 0.373 | 81.4% | Recommended |
| Rituximab | Yes | 0.373 | 81.4% | Recommended |
| Rocuronium Bromide | Yes | 0.373 | 81.4% | Recommended |
| Romidepsin | Yes | 0.373 | 81.4% | Recommended |
| Sacituzumab Govitecan | Yes | 0.373 | 81.4% | Recommended |
| Blinatumomab | Yes | 0.373 | 81.4% | Recommended |
| Etoposide phosphate | Yes | 0.373 | 81.4% | Recommended |
| Asparaginase Erwinia | Yes | 0.000 | 100.0% | Recommended |
| Azacitidine | Yes | 0.000 | 100.0% | Recommended |
| Bendamustine | Yes | 0.000 | 100.0% | Recommended |
| Bevacizumab | Yes | 0.000 | 100.0% | Recommended |
| Bleomycin | Yes | 0.000 | 100.0% | Recommended |
| Cabazitaxel | Yes | 0.000 | 100.0% | Recommended |
| Calcium folinate | Yes | 0.000 | 100.0% | Recommended |
| Carboplatin | Yes | 0.000 | 100.0% | Recommended |
| Cisplatin | Yes | 0.000 | 100.0% | Recommended |
| Cytarabine | Yes | 0.000 | 100.0% | Recommended |
| Daunorubicin | Yes | 0.000 | 100.0% | Recommended |
| DOBUTamine Hydrochloride | Yes | 0.000 | 100.0% | Recommended |
| DOPamine Hydrochloride | Yes | 0.000 | 100.0% | Recommended |
| Doxorubicin | Yes | 0.000 | 100.0% | Recommended |
| Eribulin | Yes | 0.000 | 100.0% | Recommended |
| Fentanyl Citrate | Yes | 0.000 | 100.0% | Recommended |
| Filgrastim | Yes | 0.000 | 100.0% | Recommended |
| Folic Acid | Yes | 0.000 | 100.0% | Recommended |
| Furosemide | Yes | 0.000 | 100.0% | Recommended |
| Gemcitabine | Yes | 0.000 | 100.0% | Recommended |
| Gentamicin Sulfate | Yes | 0.000 | 100.0% | Recommended |
| Heparin Sodium | Yes | 0.000 | 100.0% | Recommended |
| Idarubicin | Yes | 0.000 | 100.0% | Recommended |
| Isatuximab | Yes | 0.000 | 100.0% | Recommended |
| Levofloxacin | Yes | 0.000 | 100.0% | Recommended |
| Midazolam Hydrochloride | Yes | 0.000 | 100.0% | Recommended |
| Milrinone Lactate | Yes | 0.000 | 100.0% | Recommended |
| Mitoxantrone | Yes | 0.000 | 100.0% | Recommended |
| Nitroglycerin | Yes | 0.000 | 100.0% | Recommended |
| Oxaliplatin | Yes | 0.000 | 100.0% | Recommended |
| Propofol | Yes | 0.000 | 100.0% | Recommended |
| Alteplase | Yes | 0.373 | 81.4% | Recommended |
| Melphalan | Yes | 0.373 | 81.4% | Recommended |
| Amiodarone Hydrochloride | Yes | 0.471 | 76.4% | Recommended |
| Adrenaline (Epinephrine) | Yes | 0.745 | 62.7% | No Specific Recommendation |
| Alemtuzumab | Yes | 0.745 | 62.7% | No Specific Recommendation |
| Bicarbonate Sodium (Sodium Bicarbonate) | Yes | 0.745 | 62.7% | No Specific Recommendation |
| Bortezomib | Yes | 0.745 | 62.7% | No Specific Recommendation |
| Brentuximab Vedotin | Yes | 0.745 | 62.7% | No Specific Recommendation |
| Carmustine | Yes | 0.745 | 62.7% | No Specific Recommendation |
| Chlorphenamine | Yes | 0.745 | 62.7% | No Specific Recommendation |
| Dacarbazine | Yes | 0.745 | 62.7% | No Specific Recommendation |
| Daratumumab | Yes | 0.745 | 62.7% | No Specific Recommendation |
| Daunorubicin liposomal | Yes | 0.745 | 62.7% | No Specific Recommendation |
| Diazepam | Yes | 0.745 | 62.7% | No Specific Recommendation |
| Diphenhydramine | Yes | 0.745 | 62.7% | No Specific Recommendation |
| Doxorubicin liposomal | Yes | 0.745 | 62.7% | No Specific Recommendation |
| Enfortumab vedotin-ejfv | Yes | 0.745 | 62.7% | No Specific Recommendation |
| Gemtuzumab ozogamicin | Yes | 0.745 | 62.7% | No Specific Recommendation |
| Infliximab | Yes | 0.745 | 62.7% | No Specific Recommendation |
| Inotuzumab ozogamici | Yes | 0.745 | 62.7% | No Specific Recommendation |
| Insulin Regular | Yes | 0.745 | 62.7% | No Specific Recommendation |
| Ipilimumab | Yes | 0.745 | 62.7% | No Specific Recommendation |
| Lenograstim | Yes | 0.745 | 62.7% | No Specific Recommendation |
| Leucovorin (calcium folinate) | Yes | 0.745 | 62.7% | No Specific Recommendation |
| Magnesium sulphate | Yes | 0.745 | 62.7% | No Specific Recommendation |
| Mannitol | Yes | 0.745 | 62.7% | No Specific Recommendation |
| Mogamulizumab | Yes | 0.745 | 62.7% | No Specific Recommendation |
| Nimodipine | Yes | 0.745 | 62.7% | No Specific Recommendation |
| Nitroprusside (Sodium) | Yes | 0.745 | 62.7% | No Specific Recommendation |
| Nivolumab | Yes | 0.745 | 62.7% | No Specific Recommendation |
| Ofatumumab | Yes | 0.745 | 62.7% | No Specific Recommendation |
| Paclitaxel | Yes | 0.745 | 62.7% | No Specific Recommendation |
| Panitumumab | Yes | 0.745 | 62.7% | No Specific Recommendation |
| Polatuzumab | Yes | 0.745 | 62.7% | No Specific Recommendation |
| Pralatrexate | Yes | 0.745 | 62.7% | No Specific Recommendation |
| Ramucirumab | Yes | 0.745 | 62.7% | No Specific Recommendation |
| Remifentanil Hydrochloride | Yes | 0.745 | 62.7% | No Specific Recommendation |
| Tafasitamab-cxix | Yes | 0.745 | 62.7% | No Specific Recommendation |
| Tagraxofusp | Yes | 0.745 | 62.7% | No Specific Recommendation |
| Trastuzumab | Yes | 0.745 | 62.7% | No Specific Recommendation |
| Treosulfan | Yes | 0.745 | 62.7% | No Specific Recommendation |
| Zoledronic Acid | Yes | 0.745 | 62.7% | No Specific Recommendation |
| Ibritumomab | Yes | 0.764 | 61.8% | No Specific Recommendation |
| Fotemustine | Yes | 1.528 | 23.6% | No Specific Recommendation |
| Siltuximab | Yes | 1.528 | 23.6% | No Specific Recommendation |
| Aflibercept | Yes | 0.943 | 52.9% | No Specific Recommendation |
| Arsenic Trioxide | Yes | 0.943 | 52.9% | No Specific Recommendation |
| Belinostat | Yes | 0.943 | 52.9% | No Specific Recommendation |
| Cemiplimab | Yes | 0.943 | 52.9% | No Specific Recommendation |
| Cladribine | Yes | 0.943 | 52.9% | No Specific Recommendation |
| Copanlisib | Yes | 0.943 | 52.9% | No Specific Recommendation |
| Dactinomycin | Yes | 0.943 | 52.9% | No Specific Recommendation |
| Daunorubicin- Cytarabine liposomal | Yes | 0.943 | 52.9% | No Specific Recommendation |
| Digoxin | Yes | 0.943 | 52.9% | No Specific Recommendation |
| Dinutuximab | Yes | 0.943 | 52.9% | No Specific Recommendation |
| Disodium folinate | Yes | 0.943 | 52.9% | No Specific Recommendation |
| Durvalumab | Yes | 0.943 | 52.9% | No Specific Recommendation |
| Esmolol Hydrochloride | Yes | 0.943 | 52.9% | No Specific Recommendation |
| Irinotecan liposomal | Yes | 0.943 | 52.9% | No Specific Recommendation |
| Mechlorethamine | Yes | 0.943 | 52.9% | No Specific Recommendation |
| Naxitamab-gqgk | Yes | 0.943 | 52.9% | No Specific Recommendation |
| Necitumumab | Yes | 0.943 | 52.9% | No Specific Recommendation |
| Nelarabine | Yes | 0.943 | 52.9% | No Specific Recommendation |
| Pamidronate | Yes | 0.943 | 52.9% | No Specific Recommendation |
| Pentostatin | Yes | 0.943 | 52.9% | No Specific Recommendation |
| Procainamide Hydrochloride | Yes | 0.943 | 52.9% | No Specific Recommendation |
| Raltitrexed | Yes | 0.943 | 52.9% | No Specific Recommendation |
| Rifampicin | Yes | 0.943 | 52.9% | No Specific Recommendation |
| Streptozocin | Yes | 0.943 | 52.9% | No Specific Recommendation |
| Vinorelbine | Yes | 0.943 | 52.9% | No Specific Recommendation |
| Erlotynib | Yes | 0.980 | 51.0% | No Specific Recommendation |
| Amsacrine | Yes | 1.000 | 50.0% | No Specific Recommendation |
| Cimetidine | Yes | 1.000 | 50.0% | No Specific Recommendation |
| Belantamab mafodotin | Yes | 1.118 | 44.1% | No Specific Recommendation |
| Teicoplanin | Yes | 1.118 | 44.1% | No Specific Recommendation |
| Trastuzumab deruxtecan | Yes | 1.213 | 39.3% | No Specific Recommendation |
| Docetaxel | Yes | 1.491 | 25.5% | No Specific Recommendation |
| Etoposide | Yes | 1.491 | 25.5% | No Specific Recommendation |
| Interferon Alfa2b | Yes | 1.491 | 25.5% | No Specific Recommendation |
| Labetalol Hydrochloride | Yes | 1.491 | 25.5% | No Specific Recommendation |
| Aldesleukin (Interleukin-2) | Yes | 1.528 | 23.6% | No Specific Recommendation |
| Interferon alfa-2a | Yes | 1.528 | 23.6% | No Specific Recommendation |
| Lutetium (177Lu) Oxodotreotide | Yes | 1.528 | 23.6% | No Specific Recommendation |
| Radium 223 dichloride | Yes | 1.528 | 23.6% | No Specific Recommendation |

| **In-line Filtration** | | | | |
| --- | --- | --- | --- | --- |
| **Medication** | **Statement: Micron Filter** | **SD** | **Score Align** | **Consensus Opinion** |
| Acetylcysteine | Not Required | 0 | 100.00% | Recommended |
| Adrenaline (Epinephrine) | Not Required | 0 | 100.00% | Recommended |
| Aldesleukin (Interleukin-2) | Not Required | 0 | 100.00% | Recommended |
| Alteplase | Not Required | 0 | 100.00% | Recommended |
| Amsacrine | Not Required | 0 | 100.00% | Recommended |
| Arsenic Trioxide | Not Required | 0 | 100.00% | Recommended |
| Azacitidine | Not Required | 0 | 100.00% | Recommended |
| Belantamab mafodotin | Not Required | 0 | 100.00% | Recommended |
| Bendamustine | Not Required | 0 | 100.00% | Recommended |
| Bicarbonate Sodium (Sodium Bicarbonate) | Not Required | 0 | 100.00% | Recommended |
| Brentuximab Vedotin | Not Required | 0 | 100.00% | Recommended |
| Busulfan | Not Required | 0 | 100.00% | Recommended |
| Carfilzomib | Not Required | 0 | 100.00% | Recommended |
| Chlorphenamine | Not Required | 0 | 100.00% | Recommended |
| Cimetidine | Not Required | 0 | 100.00% | Recommended |
| Cisplatin | Not Required | 0 | 100.00% | Recommended |
| Cladribine | Not Required | 0 | 100.00% | Recommended |
| Copanlisib | Not Required | 0 | 100.00% | Recommended |
| Cyclophosphamide | Not Required | 0 | 100.00% | Recommended |
| Cytarabine | Not Required | 0 | 100.00% | Recommended |
| Dacarbazine | Not Required | 0 | 100.00% | Recommended |
| Dactinomycin | Not Required | 0 | 100.00% | Recommended |
| Decitabine | Not Required | 0 | 100.00% | Recommended |
| Denileukin Diftitox | Not Required | 0 | 100.00% | Recommended |
| Dexamethasone | Not Required | 0 | 100.00% | Recommended |
| Disodium folinate | Not Required | 0 | 100.00% | Recommended |
| DOBUTamine Hydrochloride | Not Required | 0 | 100.00% | Recommended |
| Docetaxel | Not Required | 0 | 100.00% | Recommended |
| DOPamine Hydrochloride | Not Required | 0 | 100.00% | Recommended |
| Doxorubicin | Not Required | 0 | 100.00% | Recommended |
| Doxorubicin liposomal | Not Required | 0 | 100.00% | Recommended |
| Epirubicin | Not Required | 0 | 100.00% | Recommended |
| Eribulin | Not Required | 0 | 100.00% | Recommended |
| Erlotynib | Not Required | 0 | 100.00% | Recommended |
| Esmolol Hydrochloride | Not Required | 0 | 100.00% | Recommended |
| Etoposide | Not Required | 0 | 100.00% | Recommended |
| Etoposide phosphate | Not Required | 0 | 100.00% | Recommended |
| Fentanyl Citrate | Not Required | 0 | 100.00% | Recommended |
| Filgrastim | Not Required | 0 | 100.00% | Recommended |
| Fludarabine | Not Required | 0 | 100.00% | Recommended |
| Fluorouracil (5-FU) | Not Required | 0 | 100.00% | Recommended |
| Folic Acid | Not Required | 0 | 100.00% | Recommended |
| Furosemide | Not Required | 0 | 100.00% | Recommended |
| Gemcitabine | Not Required | 0 | 100.00% | Recommended |
| Gentamicin Sulfate | Not Required | 0 | 100.00% | Recommended |
| Heparin Sodium | Not Required | 0 | 100.00% | Recommended |
| Idarubicin | Not Required | 0 | 100.00% | Recommended |
| Ifosfamide | Not Required | 0 | 100.00% | Recommended |
| Inotuzumab ozogamici | Not Required | 0 | 100.00% | Recommended |
| Insulin Regular | Not Required | 0 | 100.00% | Recommended |
| Interferon alfa-2a | Not Required | 0 | 100.00% | Recommended |
| Interferon Alfa2b | Not Required | 0 | 100.00% | Recommended |
| Irinotecan hydrchloride | Not Required | 0 | 100.00% | Recommended |
| Irinotecan liposomal | Not Required | 0 | 100.00% | Recommended |
| Labetalol Hydrochloride | Not Required | 0 | 100.00% | Recommended |
| Lenograstim | Not Required | 0 | 100.00% | Recommended |
| Leucovorin (calcium folinate) | Not Required | 0 | 100.00% | Recommended |
| Levofloxacin | Not Required | 0 | 100.00% | Recommended |
| Lutetium (177Lu) Oxodotreotide | Not Required | 0 | 100.00% | Recommended |
| Mechlorethamine | Not Required | 0 | 100.00% | Recommended |
| Melphalan | Not Required | 0 | 100.00% | Recommended |
| Meropenem | Not Required | 0 | 100.00% | Recommended |
| Mesna | Not Required | 0 | 100.00% | Recommended |
| Methotrexate | Not Required | 0 | 100.00% | Recommended |
| Methylprednisolone sodium succinate | Not Required | 0 | 100.00% | Recommended |
| Midazolam Hydrochloride | Not Required | 0 | 100.00% | Recommended |
| Milrinone Lactate | Not Required | 0 | 100.00% | Recommended |
| Mitomycin | Not Required | 0 | 100.00% | Recommended |
| Mitoxantrone | Not Required | 0 | 100.00% | Recommended |
| Morphine Hydrochloride | Not Required | 0 | 100.00% | Recommended |
| Naxitamab-gqgk | Not Required | 0 | 100.00% | Recommended |
| Necitumumab | Not Required | 0 | 100.00% | Recommended |
| Nelarabine | Not Required | 0 | 100.00% | Recommended |
| Nimodipine | Not Required | 0 | 100.00% | Recommended |
| Nitroglycerin | Not Required | 0 | 100.00% | Recommended |
| Nitroprusside (Sodium) | Not Required | 0 | 100.00% | Recommended |
| Noradrenaline Acid Tartrate / Norepinephrine | Not Required | 0 | 100.00% | Recommended |
| Obinutuzumab | Not Required | 0 | 100.00% | Recommended |
| Ofatumumab | Not Required | 0 | 100.00% | Recommended |
| Omeprazole Sodium | Not Required | 0 | 100.00% | Recommended |
| Ondansetron Hydrochloride | Not Required | 0 | 100.00% | Recommended |
| Oxaliplatin | Not Required | 0 | 100.00% | Recommended |
| Pamidronate | Not Required | 0 | 100.00% | Recommended |
| Paracetamol | Not Required | 0 | 100.00% | Recommended |
| Pegaspargase | Not Required | 0 | 100.00% | Recommended |
| Pemetrexed | Not Required | 0 | 100.00% | Recommended |
| Pentostatin | Not Required | 0 | 100.00% | Recommended |
| Pertuzumab | Not Required | 0 | 100.00% | Recommended |
| Piperacillin Sodium-Tazobactam Sodium | Not Required | 0 | 100.00% | Recommended |
| Potassium Chloride | Not Required | 0 | 100.00% | Recommended |
| Pralatrexate | Not Required | 0 | 100.00% | Recommended |
| Procainamide Hydrochloride | Not Required | 0 | 100.00% | Recommended |
| Propofol | Not Required | 0 | 100.00% | Recommended |
| Radium 223 dichloride | Not Required | 0 | 100.00% | Recommended |
| Raltitrexed | Not Required | 0 | 100.00% | Recommended |
| Ranitidine | Not Required | 0 | 100.00% | Recommended |
| Remifentanil Hydrochloride | Not Required | 0 | 100.00% | Recommended |
| Rifampicin | Not Required | 0 | 100.00% | Recommended |
| Rituximab | Not Required | 0 | 100.00% | Recommended |
| Rocuronium Bromide | Not Required | 0 | 100.00% | Recommended |
| Romidepsin | Not Required | 0 | 100.00% | Recommended |
| Sacituzumab Govitecan | Not Required | 0 | 100.00% | Recommended |
| Salbutamol Sulfate | Not Required | 0 | 100.00% | Recommended |
| Streptozocin | Not Required | 0 | 100.00% | Recommended |
| Tafasitamab-cxix | Not Required | 0 | 100.00% | Recommended |
| Teicoplanin | Not Required | 0 | 100.00% | Recommended |
| Tocilizumab | Not Required | 0 | 100.00% | Recommended |
| Topotecan | Not Required | 0 | 100.00% | Recommended |
| Trastuzumab | Not Required | 0 | 100.00% | Recommended |
| Treosulfan | Not Required | 0 | 100.00% | Recommended |
| Vancomycin Hydrochloride | Not Required | 0 | 100.00% | Recommended |
| Vecuronium Bromide | Not Required | 0 | 100.00% | Recommended |
| Vinblastine | Not Required | 0 | 100.00% | Recommended |
| Vincristine | Not Required | 0 | 100.00% | Recommended |
| Vindesine | Not Required | 0 | 100.00% | Recommended |
| Vinflunine | Not Required | 0 | 100.00% | Recommended |
| Vinorelbine | Not Required | 0 | 100.00% | Recommended |
| Zoledronic Acid | Not Required | 0 | 100.00% | Recommended |
| Amiodarone Hydrochloride | Not Required | 0.373 | 81.40% | Recommended |
| Bevacizumab | Not Required | 0.373 | 81.40% | Recommended |
| Bleomycin | Not Required | 0.373 | 81.40% | Recommended |
| Bortezomib | Not Required | 0.373 | 81.40% | Recommended |
| Calcium folinate | Not Required | 0.373 | 81.40% | Recommended |
| Carboplatin | Not Required | 0.373 | 81.40% | Recommended |
| Carmustine | Not Required | 0.373 | 81.40% | Recommended |
| Daunorubicin | Not Required | 0.373 | 81.40% | Recommended |
| Diazepam | Not Required | 0.373 | 81.40% | Recommended |
| Digoxin | Not Required | 0.373 | 81.40% | Recommended |
| Diphenhydramine | Not Required | 0.373 | 81.40% | Recommended |
| Enfortumab vedotin-ejfv | Not Required | 0.373 | 81.40% | Recommended |
| Alemtuzumab | Not Required | 0.373 | 81.40% | Recommended |
| Pembrolizumab | 0.2-5 | 0 | 100.00% | Recommended |
| Ipilimumab | 0.2-1.2 | 0.373 | 81.40% | Recommended |
| Temsirolimus | 1.2 | 0 | 100.00% | Recommended |
| Infliximab | 1.2 | 0.373 | 81.40% | Recommended |
| Gemtuzumab ozogamicin | 0.2 | 0 | 100.00% | Recommended |
| Ibritumomab | 0.2 | 0 | 100.00% | Recommended |
| Margetuximab-cmkb | 0.2 | 0 | 100.00% | Recommended |
| Mogamulizumab | 0.2 | 0 | 100.00% | Recommended |
| Panitumumab | 0.2 | 0 | 100.00% | Recommended |
| Polatuzumab | 0.2 | 0 | 100.00% | Recommended |
| Ramucirumab | 0.2 | 0 | 100.00% | Recommended |
| Tagraxofusp | 0.2 | 0 | 100.00% | Recommended |
| Durvalumab | 0.2 | 0 | 100.00% | Recommended |
| Trastuzumab emtansine | 0.2 | 0 | 100.00% | Recommended |
| Cemiplimab | 0.2 | 0 | 100.00% | Recommended |
| Blinatumomab | 0.2 | 0 | 100.00% | Recommended |
| Cabazitaxel | 0.2 | 0 | 100.00% | Recommended |
| Daratumumab | 0.2 | 0 | 100.00% | Recommended |
| Nivolumab | 0.2 | 0.4 | 80.00% | Recommended |
| Siltuximab | 0.2 | 0 | 100.00% | Recommended |
| Atezolizumab | Not Required | 0.764 | 61.80% | No Specific Recommendation |
| Lurbinectedin | Not Required | 1.118 | 44.10% | No Specific Recommendation |
| Asparaginase Erwinia | Not Required | 1.213 | 39.30% | No Specific Recommendation |
| L-asparaginase | Not Required | 1.491 | 25.50% | No Specific Recommendation |
| Paclitaxel Albumin bound | Not Required | 1.528 | 23.60% | No Specific Recommendation |
| Belinostat | Not Required | 1.795 | 10.20% | No Specific Recommendation |
| Aflibercept | Not Required | 1.886 | 5.70% | No Specific Recommendation |
| Trabectedin | Not Required | 1.886 | 5.70% | No Specific Recommendation |
| Daunorubicin- Cytarabine liposomal | 15 | 0.943 | 52.90% | No Specific Recommendation |
| Daunorubicin liposomal | 15 | 1.795 | 10.20% | No Specific Recommendation |
| Elotuzumab | 0.2-1.2 | 0.745 | 62.70% | No Specific Recommendation |
| Mannitol | 0.2/1.2 | 1.607 | 19.60% | No Specific Recommendation |
| Asparaginase (E. coli) | 1.2 | 0.898 | 55.10% | No Specific Recommendation |
| Trastuzumab deruxtecan | 0.2 | 0.696 | 65.20% | No Specific Recommendation |
| Avelumab | 0.2 | 0.745 | 62.70% | No Specific Recommendation |
| Isatuximab | 0.2 | 0.745 | 62.70% | No Specific Recommendation |
| Paclitaxel | 0.2 | 0.745 | 62.70% | No Specific Recommendation |
| Thiotepa | 0.2 | 0.745 | 62.70% | No Specific Recommendation |
| Clofarabine | 0.2 | 1 | 50.00% | No Specific Recommendation |
| Dinutuximab | 0.2 | 1.462 | 26.90% | No Specific Recommendation |
| Cetuximab | 0.2 | 1.491 | 25.50% | No Specific Recommendation |
| Fotemustine | 0.2 | 1.528 | 23.60% | No Specific Recommendation |
| Magnesium sulphate | 0.2 | 1.528 | 23.60% | No Specific Recommendation |

| **Light Protection During Storage** | | | | |
| --- | --- | --- | --- | --- |
| **Medication** | **Statement: Light Protection** | **SD** | **Score Align** | **Consensus Opinion** |
| Filgrastim | Yes | 0 | 100.00% | Recommended |
| Siltuximab | Yes | 0 | 100.00% | Recommended |
| Fotemustine | Yes | 0 | 100.00% | Recommended |
| Adrenaline (Epinephrine) | Yes | 0 | 100.00% | Recommended |
| Alteplase | Yes | 0 | 100.00% | Recommended |
| Blinatumomab | Yes | 0 | 100.00% | Recommended |
| Cemiplimab | Yes | 0 | 100.00% | Recommended |
| Chlorphenamine | Yes | 0 | 100.00% | Recommended |
| Cisplatin | Yes | 0 | 100.00% | Recommended |
| Dacarbazine | Yes | 0 | 100.00% | Recommended |
| Daratumumab | Yes | 0 | 100.00% | Recommended |
| Docetaxel | Yes | 0 | 100.00% | Recommended |
| DOPamine Hydrochloride | Yes | 0 | 100.00% | Recommended |
| Doxorubicin | Yes | 0 | 100.00% | Recommended |
| Epirubicin | Yes | 0 | 100.00% | Recommended |
| Etoposide phosphate | Yes | 0 | 100.00% | Recommended |
| Fentanyl Citrate | Yes | 0 | 100.00% | Recommended |
| Fluorouracil (5-FU) | Yes | 0 | 100.00% | Recommended |
| Folic Acid | Yes | 0 | 100.00% | Recommended |
| Furosemide | Yes | 0 | 100.00% | Recommended |
| Idarubicin | Yes | 0 | 100.00% | Recommended |
| Inotuzumab ozogamici | Yes | 0 | 100.00% | Recommended |
| Ipilimumab | Yes | 0 | 100.00% | Recommended |
| Irinotecan hydrchloride | Yes | 0 | 100.00% | Recommended |
| Irinotecan liposomal | Yes | 0 | 100.00% | Recommended |
| Leucovorin (calcium folinate) | Yes | 0 | 100.00% | Recommended |
| Mechlorethamine | Yes | 0 | 100.00% | Recommended |
| Melphalan | Yes | 0 | 100.00% | Recommended |
| Methotrexate | Yes | 0 | 100.00% | Recommended |
| Mitomycin | Yes | 0 | 100.00% | Recommended |
| Naxitamab-gqgk | Yes | 0 | 100.00% | Recommended |
| Necitumumab | Yes | 0 | 100.00% | Recommended |
| Nitroprusside (Sodium) | Yes | 0 | 100.00% | Recommended |
| Noradrenaline Acid Tartrate / Norepinephrine | Yes | 0 | 100.00% | Recommended |
| Ondansetron Hydrochloride | Yes | 0 | 100.00% | Recommended |
| Oxaliplatin | Yes | 0 | 100.00% | Recommended |
| Paclitaxel | Yes | 0 | 100.00% | Recommended |
| Paclitaxel Albumin bound | Yes | 0 | 100.00% | Recommended |
| Panitumumab | Yes | 0 | 100.00% | Recommended |
| Pertuzumab | Yes | 0 | 100.00% | Recommended |
| Pralatrexate | Yes | 0 | 100.00% | Recommended |
| Ramucirumab | Yes | 0 | 100.00% | Recommended |
| Ranitidine | Yes | 0 | 100.00% | Recommended |
| Sacituzumab Govitecan | Yes | 0 | 100.00% | Recommended |
| Salbutamol Sulfate | Yes | 0 | 100.00% | Recommended |
| Tafasitamab-cxix | Yes | 0 | 100.00% | Recommended |
| Tagraxofusp | Yes | 0 | 100.00% | Recommended |
| Temsirolimus | Yes | 0 | 100.00% | Recommended |
| Thiotepa | Yes | 0 | 100.00% | Recommended |
| Topotecan | Yes | 0 | 100.00% | Recommended |
| Trastuzumab deruxtecan | Yes | 0 | 100.00% | Recommended |
| Vinblastine | Yes | 0 | 100.00% | Recommended |
| Vincristine | Yes | 0 | 100.00% | Recommended |
| Atezolizumab | Yes | 0.373 | 81.37% | Recommended |
| Bevacizumab | Yes | 0.373 | 81.37% | Recommended |
| Bleomycin | Yes | 0.373 | 81.37% | Recommended |
| Bortezomib | Yes | 0.373 | 81.37% | Recommended |
| Calcium folinate | Yes | 0.373 | 81.37% | Recommended |
| Carboplatin | Yes | 0.373 | 81.37% | Recommended |
| Carmustine | Yes | 0.373 | 81.37% | Recommended |
| Dactinomycin | Yes | 0.373 | 81.37% | Recommended |
| Daunorubicin | Yes | 0.373 | 81.37% | Recommended |
| Daunorubicin- Cytarabine liposomal | Yes | 0.373 | 81.37% | Recommended |
| Dexamethasone | Yes | 0.373 | 81.37% | Recommended |
| Diazepam | Yes | 0.373 | 81.37% | Recommended |
| Digoxin | Yes | 0.373 | 81.37% | Recommended |
| Diphenhydramine | Yes | 0.373 | 81.37% | Recommended |
| Methylprednisolone sodium succinate | Yes | 0.373 | 81.37% | Recommended |
| Vinflunine | Yes | 0.373 | 81.37% | Recommended |
| Aldesleukin (Interleukin-2) | Yes | 0 | 100.00% | Recommended |
| Alemtuzumab | Yes | 0 | 100.00% | Recommended |
| Daunorubicin liposomal | Yes | 0 | 100.00% | Recommended |
| Interferon alfa-2a | Yes | 0 | 100.00% | Recommended |
| Morphine Hydrochloride | Yes | 0 | 100.00% | Recommended |
| Rituximab | Yes | 0 | 100.00% | Recommended |
| Vinorelbine | Yes | 0 | 100.00% | Recommended |
| Arsenic Trioxide | Not Required | 0 | 100.00% | Recommended |
| Belantamab mafodotin | Not Required | 0 | 100.00% | Recommended |
| Bicarbonate Sodium (Sodium Bicarbonate) | Not Required | 0 | 100.00% | Recommended |
| Busulfan | Not Required | 0 | 100.00% | Recommended |
| Cabazitaxel | Not Required | 0 | 100.00% | Recommended |
| Cetuximab | Not Required | 0 | 100.00% | Recommended |
| Denileukin Diftitox | Not Required | 0 | 100.00% | Recommended |
| Erlotynib | Not Required | 0 | 100.00% | Recommended |
| Esmolol Hydrochloride | Not Required | 0 | 100.00% | Recommended |
| Gemcitabine | Not Required | 0 | 100.00% | Recommended |
| Gentamicin Sulfate | Not Required | 0 | 100.00% | Recommended |
| Lenograstim | Not Required | 0 | 100.00% | Recommended |
| Magnesium sulphate | Not Required | 0 | 100.00% | Recommended |
| Nelarabine | Not Required | 0 | 100.00% | Recommended |
| Pamidronate | Not Required | 0 | 100.00% | Recommended |
| Piperacillin Sodium-Tazobactam Sodium | Not Required | 0 | 100.00% | Recommended |
| Radium 223 dichloride | Not Required | 0 | 100.00% | Recommended |
| Rocuronium Bromide | Not Required | 0 | 100.00% | Recommended |
| Teicoplanin | Not Required | 0 | 100.00% | Recommended |
| Trabectedin | Not Required | 0 | 100.00% | Recommended |
| Treosulfan | Not Required | 0 | 100.00% | Recommended |
| Vancomycin Hydrochloride | Not Required | 0 | 100.00% | Recommended |
| Zoledronic Acid | Not Required | 0 | 100.00% | Recommended |
| Copanlisib | Not Required | 0.373 | 81.37% | Recommended |
| Milrinone Lactate | Not Required | 0.373 | 81.37% | Recommended |
| Mannitol | Not Required | 0 | 100.00% | Recommended |
| Acetylcysteine | Yes | 0.745 | 62.73% | No Specific Recommendation |
| Asparaginase Erwinia | Yes | 0.745 | 62.73% | No Specific Recommendation |
| Bendamustine | Yes | 0.745 | 62.73% | No Specific Recommendation |
| Brentuximab Vedotin | Yes | 0.745 | 62.73% | No Specific Recommendation |
| Cladribine | Yes | 0.745 | 62.73% | No Specific Recommendation |
| Cytarabine | Yes | 0.745 | 62.73% | No Specific Recommendation |
| Insulin Regular | Yes | 0.745 | 62.73% | No Specific Recommendation |
| Midazolam Hydrochloride | Yes | 0.745 | 62.73% | No Specific Recommendation |
| Pegaspargase | Yes | 0.745 | 62.73% | No Specific Recommendation |
| DOBUTamine Hydrochloride | Yes | 0.696 | 65.20% | No Specific Recommendation |
| Amiodarone Hydrochloride | Yes | 0.661 | 67.00% | No Specific Recommendation |
| Paracetamol | Yes | 0.764 | 61.81% | No Specific Recommendation |
| Rifampicin | Yes | 1.118 | 44.10% | No Specific Recommendation |
| Vecuronium Bromide | Yes | 1.118 | 44.10% | No Specific Recommendation |
| Vindesine | Yes | 1.118 | 44.10% | No Specific Recommendation |
| Azacitidine | Yes | 1.374 | 31.28% | No Specific Recommendation |
| Asparaginase (E. coli) | Yes | 1.491 | 25.46% | No Specific Recommendation |
| Etoposide | Yes | 1.491 | 25.46% | No Specific Recommendation |
| Labetalol Hydrochloride | Yes | 1.491 | 25.46% | No Specific Recommendation |
| Levofloxacin | Yes | 1.491 | 25.46% | No Specific Recommendation |
| Nimodipine | Yes | 1.491 | 25.46% | No Specific Recommendation |
| Nitroglycerin | Yes | 1.491 | 25.46% | No Specific Recommendation |
| Raltitrexed | Yes | 1.491 | 25.46% | No Specific Recommendation |
| Streptozocin | Yes | 1.491 | 25.46% | No Specific Recommendation |
| Decitabine | Yes | 1.528 | 23.62% | No Specific Recommendation |
| Interferon Alfa2b | Yes | 1.528 | 23.62% | No Specific Recommendation |
| Omeprazole Sodium | Yes | 1.528 | 23.62% | No Specific Recommendation |
| Procainamide Hydrochloride | Yes | 1.528 | 23.62% | No Specific Recommendation |
| Trastuzumab emtansine | Yes | 1.572 | 21.38% | No Specific Recommendation |
| Doxorubicin liposomal | Yes | 1.599 | 20.07% | No Specific Recommendation |
| Eribulin | Yes | 1.675 | 16.25% | No Specific Recommendation |
| Remifentanil Hydrochloride | Yes | 1.675 | 16.25% | No Specific Recommendation |
| Clofarabine | Yes | 1.795 | 10.25% | No Specific Recommendation |
| Mitoxantrone | Yes | 1.795 | 10.25% | No Specific Recommendation |
| Amsacrine | Yes | 1.691 | 15.50% | No Specific Recommendation |
| Heparin Sodium | Yes | 1.886 | 5.72% | No Specific Recommendation |
| L-asparaginase | Yes | 1.886 | 5.72% | No Specific Recommendation |
| Mesna | Yes | 1.886 | 5.72% | No Specific Recommendation |
| Pentostatin | Yes | 1.886 | 5.72% | No Specific Recommendation |
| Potassium Chloride | Yes | 1.886 | 5.72% | No Specific Recommendation |
| Meropenem | Yes | 2 | 0.00% | No Specific Recommendation |
| Lutetium (177Lu) Oxodotreotide | Not Required | 0.745 | 62.73% | No Specific Recommendation |
| Lurbinectedin | Not Required | 0.943 | 52.86% | No Specific Recommendation |
| Romidepsin | Not Required | 1.106 | 44.72% | No Specific Recommendation |
| Ifosfamide | Not Required | 1.118 | 44.10% | No Specific Recommendation |
| Enfortumab vedotin-ejfv | Not Required | 1.374 | 31.28% | No Specific Recommendation |
| Cimetidine | Not Required | 1.462 | 26.88% | No Specific Recommendation |
| Propofol | Not Required | 1.462 | 26.88% | No Specific Recommendation |
| Aflibercept | Not Required | 1.491 | 25.46% | No Specific Recommendation |
| Fludarabine | Not Required | 1.491 | 25.46% | No Specific Recommendation |
| Polatuzumab | Not Required | 1.491 | 25.46% | No Specific Recommendation |
| Pembrolizumab | Not Required | 1.528 | 23.62% | No Specific Recommendation |
| Pemetrexed | Not Required | 1.528 | 23.62% | No Specific Recommendation |
| Trastuzumab | Not Required | 1.528 | 23.62% | No Specific Recommendation |
| Infliximab | Not Required | 1.675 | 16.25% | No Specific Recommendation |
| Belinostat | Not Required | 1.795 | 10.25% | No Specific Recommendation |
| Dinutuximab | Not Required | 1.795 | 10.25% | No Specific Recommendation |
| Isatuximab | Not Required | 1.863 | 6.83% | No Specific Recommendation |
| Avelumab | Not Required | 1.886 | 5.72% | No Specific Recommendation |
| Carfilzomib | Not Required | 1.886 | 5.72% | No Specific Recommendation |
| Cyclophosphamide | Not Required | 1.886 | 5.72% | No Specific Recommendation |
| Durvalumab | Not Required | 1.886 | 5.72% | No Specific Recommendation |
| Elotuzumab | Not Required | 1.886 | 5.72% | No Specific Recommendation |
| Gemtuzumab ozogamicin | Not Required | 1.886 | 5.72% | No Specific Recommendation |
| Ibritumomab | Not Required | 1.886 | 5.72% | No Specific Recommendation |
| Mogamulizumab | Not Required | 1.886 | 5.72% | No Specific Recommendation |
| Nivolumab | Not Required | 1.886 | 5.72% | No Specific Recommendation |
| Ofatumumab | Not Required | 1.886 | 5.72% | No Specific Recommendation |
| Disodium folinate | Not Required | 2 | 0.00% | No Specific Recommendation |
| Margetuximab-cmkb | Not Required | 2 | 0.00% | No Specific Recommendation |
| Obinutuzumab | Not Required | 2 | 0.00% | No Specific Recommendation |
| Tocilizumab | Not Required | 2 | 0.00% | No Specific Recommendation |

| **Light Protection During Administration** | | | | | | |
| --- | --- | --- | --- | --- | --- | --- |
| **Medication** | **Statement: Light Protection** | | **SD** | | **Score Align** | **Consensus Opinion** |
| Fotemustine | | Yes | | 0 | 100.00% | Recommended |
| Dacarbazine | | Yes | | 0 | 100.00% | Recommended |
| Nitroprusside (Sodium) | | Yes | | 0 | 100.00% | Recommended |
| Sacituzumab Govitecan | | Yes | | 0 | 100.00% | Recommended |
| Trastuzumab deruxtecan | | Yes | | 0 | 100.00% | Recommended |
| Inotuzumab ozogamici | | Yes | | 0.4 | 80.00% | Recommended |
| Labetalol Hydrochloride | | Yes | | 0.4 | 80.00% | Recommended |
| Temsirolimus | | Yes | | 0 | 100.00% | Recommended |
| Aflibercept | | Not Required | | 0 | 100.00% | Recommended |
| Aldesleukin (Interleukin-2) | | Not Required | | 0 | 100.00% | Recommended |
| Alteplase | | Not Required | | 0 | 100.00% | Recommended |
| Arsenic Trioxide | | Not Required | | 0 | 100.00% | Recommended |
| Asparaginase (E. coli) | | Not Required | | 0 | 100.00% | Recommended |
| Asparaginase Erwinia | | Not Required | | 0 | 100.00% | Recommended |
| Atezolizumab | | Not Required | | 0 | 100.00% | Recommended |
| Avelumab | | Not Required | | 0 | 100.00% | Recommended |
| Azacitidine | | Not Required | | 0 | 100.00% | Recommended |
| Belantamab mafodotin | | Not Required | | 0 | 100.00% | Recommended |
| Belinostat | | Not Required | | 0 | 100.00% | Recommended |
| Bicarbonate Sodium (Sodium Bicarbonate) | | Not Required | | 0 | 100.00% | Recommended |
| Busulfan | | Not Required | | 0 | 100.00% | Recommended |
| Cabazitaxel | | Not Required | | 0 | 100.00% | Recommended |
| Cemiplimab | | Not Required | | 0 | 100.00% | Recommended |
| Cetuximab | | Not Required | | 0 | 100.00% | Recommended |
| Cimetidine | | Not Required | | 0 | 100.00% | Recommended |
| Cladribine | | Not Required | | 0 | 100.00% | Recommended |
| Clofarabine | | Not Required | | 0 | 100.00% | Recommended |
| Dactinomycin | | Not Required | | 0 | 100.00% | Recommended |
| Denileukin Diftitox | | Not Required | | 0 | 100.00% | Recommended |
| Disodium folinate | | Not Required | | 0 | 100.00% | Recommended |
| DOBUTamine Hydrochloride | | Not Required | | 0 | 100.00% | Recommended |
| Docetaxel | | Not Required | | 0 | 100.00% | Recommended |
| Durvalumab | | Not Required | | 0 | 100.00% | Recommended |
| Elotuzumab | | Not Required | | 0 | 100.00% | Recommended |
| Erlotynib | | Not Required | | 0 | 100.00% | Recommended |
| Esmolol Hydrochloride | | Not Required | | 0 | 100.00% | Recommended |
| Etoposide phosphate | | Not Required | | 0 | 100.00% | Recommended |
| Fentanyl Citrate | | Not Required | | 0 | 100.00% | Recommended |
| Fludarabine | | Not Required | | 0 | 100.00% | Recommended |
| Gemcitabine | | Not Required | | 0 | 100.00% | Recommended |
| Infliximab | | Not Required | | 0 | 100.00% | Recommended |
| Ipilimumab | | Not Required | | 0 | 100.00% | Recommended |
| Isatuximab | | Not Required | | 0 | 100.00% | Recommended |
| L-asparaginase | | Not Required | | 0 | 100.00% | Recommended |
| Lenograstim | | Not Required | | 0 | 100.00% | Recommended |
| Lurbinectedin | | Not Required | | 0 | 100.00% | Recommended |
| Magnesium sulphate | | Not Required | | 0 | 100.00% | Recommended |
| Margetuximab-cmkb | | Not Required | | 0 | 100.00% | Recommended |
| Mesna | | Not Required | | 0 | 100.00% | Recommended |
| Milrinone Lactate | | Not Required | | 0 | 100.00% | Recommended |
| Mogamulizumab | | Not Required | | 0 | 100.00% | Recommended |
| Nelarabine | | Not Required | | 0 | 100.00% | Recommended |
| Nivolumab | | Not Required | | 0 | 100.00% | Recommended |
| Obinutuzumab | | Not Required | | 0 | 100.00% | Recommended |
| Ofatumumab | | Not Required | | 0 | 100.00% | Recommended |
| Ondansetron Hydrochloride | | Not Required | | 0 | 100.00% | Recommended |
| Pamidronate | | Not Required | | 0 | 100.00% | Recommended |
| Paracetamol | | Not Required | | 0 | 100.00% | Recommended |
| Pembrolizumab | | Not Required | | 0 | 100.00% | Recommended |
| Piperacillin Sodium-Tazobactam Sodium | | Not Required | | 0 | 100.00% | Recommended |
| Potassium Chloride | | Not Required | | 0 | 100.00% | Recommended |
| Pralatrexate | | Not Required | | 0 | 100.00% | Recommended |
| Procainamide Hydrochloride | | Not Required | | 0 | 100.00% | Recommended |
| Propofol | | Not Required | | 0 | 100.00% | Recommended |
| Radium 223 dichloride | | Not Required | | 0 | 100.00% | Recommended |
| Ramucirumab | | Not Required | | 0 | 100.00% | Recommended |
| Ranitidine | | Not Required | | 0 | 100.00% | Recommended |
| Remifentanil Hydrochloride | | Not Required | | 0 | 100.00% | Recommended |
| Rocuronium Bromide | | Not Required | | 0 | 100.00% | Recommended |
| Romidepsin | | Not Required | | 0 | 100.00% | Recommended |
| Salbutamol Sulfate | | Not Required | | 0 | 100.00% | Recommended |
| Streptozocin | | Not Required | | 0 | 100.00% | Recommended |
| Tagraxofusp | | Not Required | | 0 | 100.00% | Recommended |
| Teicoplanin | | Not Required | | 0 | 100.00% | Recommended |
| Trabectedin | | Not Required | | 0 | 100.00% | Recommended |
| Trastuzumab | | Not Required | | 0 | 100.00% | Recommended |
| Treosulfan | | Not Required | | 0 | 100.00% | Recommended |
| Vancomycin Hydrochloride | | Not Required | | 0 | 100.00% | Recommended |
| Vecuronium Bromide | | Not Required | | 0 | 100.00% | Recommended |
| Zoledronic Acid | | Not Required | | 0 | 100.00% | Recommended |
| Acetylcysteine | | Not Required | | 0.4 | 80.00% | Recommended |
| Copanlisib | | Not Required | | 0.4 | 80.00% | Recommended |
| Daunorubicin- Cytarabine liposomal | | Not Required | | 0.4 | 80.00% | Recommended |
| Diazepam | | Not Required | | 0.4 | 80.00% | Recommended |
| Enfortumab vedotin-ejfv | | Not Required | | 0.4 | 80.00% | Recommended |
| Heparin Sodium | | Not Required | | 0.4 | 80.00% | Recommended |
| Methylprednisolone sodium succinate | | Not Required | | 0.4 | 80.00% | Recommended |
| Pertuzumab | | Not Required | | 0.4 | 80.00% | Recommended |
| Rifampicin | | Not Required | | 0.4 | 80.00% | Recommended |
| Tafasitamab-cxix | | Not Required | | 0.4 | 80.00% | Recommended |
| Tocilizumab | | Not Required | | 0.4 | 80.00% | Recommended |
| Amsacrine | | Not Required | | 0 | 100.00% | Recommended |
| Lutetium (177Lu) Oxodotreotide | | Not Required | | 0 | 100.00% | Recommended |
| Mannitol | | Not Required | | 0 | 100.00% | Recommended |
| Omeprazole Sodium | | Not Required | | 0 | 100.00% | Recommended |
| Ibritumomab | | Not Required | | 0.373 | 81.37% | Recommended |
| Leucovorin (calcium folinate) | | Yes | | 0.764 | 61.81% | No Specific Recommendation |
| Siltuximab | | Yes | | 2 | 0.00% | No Specific Recommendation |
| Filgrastim | | Yes | | 1.213 | 39.33% | No Specific Recommendation |
| Necitumumab | | Yes | | 1.213 | 39.33% | No Specific Recommendation |
| Decitabine | | Yes | | 1.258 | 37.08% | No Specific Recommendation |
| Levofloxacin | | Yes | | 1.374 | 31.28% | No Specific Recommendation |
| Naxitamab-gqgk | | Yes | | 1.374 | 31.28% | No Specific Recommendation |
| Daunorubicin | | Yes | | 1.414 | 29.29% | No Specific Recommendation |
| Bendamustine | | Yes | | 1.462 | 26.88% | No Specific Recommendation |
| Doxorubicin | | Yes | | 1.462 | 26.88% | No Specific Recommendation |
| Irinotecan liposomal | | Yes | | 1.462 | 26.88% | No Specific Recommendation |
| Nimodipine | | Yes | | 1.462 | 26.88% | No Specific Recommendation |
| Adrenaline (Epinephrine) | | Yes | | 1.491 | 25.46% | No Specific Recommendation |
| Bleomycin | | Yes | | 1.491 | 25.46% | No Specific Recommendation |
| Brentuximab Vedotin | | Yes | | 1.491 | 25.46% | No Specific Recommendation |
| Carmustine | | Yes | | 1.491 | 25.46% | No Specific Recommendation |
| Chlorphenamine | | Yes | | 1.491 | 25.46% | No Specific Recommendation |
| Cisplatin | | Yes | | 1.491 | 25.46% | No Specific Recommendation |
| Doxorubicin liposomal | | Yes | | 1.491 | 25.46% | No Specific Recommendation |
| Idarubicin | | Yes | | 1.491 | 25.46% | No Specific Recommendation |
| Interferon alfa-2a | | Yes | | 1.491 | 25.46% | No Specific Recommendation |
| Interferon Alfa2b | | Yes | | 1.491 | 25.46% | No Specific Recommendation |
| Bevacizumab | | Yes | | 1.528 | 23.62% | No Specific Recommendation |
| Mitoxantrone | | Yes | | 1.528 | 23.62% | No Specific Recommendation |
| Bortezomib | | Yes | | 1.607 | 19.64% | No Specific Recommendation |
| Epirubicin | | Yes | | 1.607 | 19.64% | No Specific Recommendation |
| Panitumumab | | Yes | | 1.607 | 19.64% | No Specific Recommendation |
| Eribulin | | Yes | | 1.633 | 18.35% | No Specific Recommendation |
| Alemtuzumab | | Yes | | 1.675 | 16.25% | No Specific Recommendation |
| Morphine Hydrochloride | | Yes | | 1.675 | 16.25% | No Specific Recommendation |
| Trastuzumab emtansine | | Yes | | 1.675 | 16.25% | No Specific Recommendation |
| Paclitaxel Albumin bound | | Yes | | 1.732 | 13.40% | No Specific Recommendation |
| Digoxin | | Yes | | 1.795 | 10.25% | No Specific Recommendation |
| Diphenhydramine | | Yes | | 1.795 | 10.25% | No Specific Recommendation |
| Furosemide | | Yes | | 1.795 | 10.25% | No Specific Recommendation |
| Meropenem | | Yes | | 1.795 | 10.25% | No Specific Recommendation |
| Noradrenaline Acid Tartrate / Norepinephrine | | Yes | | 1.795 | 10.25% | No Specific Recommendation |
| Oxaliplatin | | Yes | | 1.795 | 10.25% | No Specific Recommendation |
| Thiotepa | | Yes | | 1.795 | 10.25% | No Specific Recommendation |
| Topotecan | | Yes | | 1.795 | 10.25% | No Specific Recommendation |
| Amiodarone Hydrochloride | | Yes | | 1.803 | 9.86% | No Specific Recommendation |
| Calcium folinate | | Yes | | 1.803 | 9.86% | No Specific Recommendation |
| Cytarabine | | Yes | | 1.803 | 9.86% | No Specific Recommendation |
| Irinotecan hydrchloride | | Yes | | 1.803 | 9.86% | No Specific Recommendation |
| Raltitrexed | | Yes | | 1.803 | 9.86% | No Specific Recommendation |
| Rituximab | | Yes | | 1.803 | 9.86% | No Specific Recommendation |
| Vincristine | | Yes | | 1.803 | 9.86% | No Specific Recommendation |
| Vindesine | | Yes | | 1.803 | 9.86% | No Specific Recommendation |
| Carboplatin | | Yes | | 1.863 | 6.83% | No Specific Recommendation |
| Vinflunine | | Yes | | 1.863 | 6.83% | No Specific Recommendation |
| Vinorelbine | | Yes | | 1.863 | 6.83% | No Specific Recommendation |
| Blinatumomab | | Yes | | 1.886 | 5.72% | No Specific Recommendation |
| Fluorouracil (5-FU) | | Yes | | 1.886 | 5.72% | No Specific Recommendation |
| Gentamicin Sulfate | | Yes | | 1.886 | 5.72% | No Specific Recommendation |
| Mechlorethamine | | Yes | | 1.886 | 5.72% | No Specific Recommendation |
| Methotrexate | | Yes | | 1.886 | 5.72% | No Specific Recommendation |
| Midazolam Hydrochloride | | Yes | | 1.886 | 5.72% | No Specific Recommendation |
| Mitomycin | | Yes | | 1.886 | 5.72% | No Specific Recommendation |
| Pegaspargase | | Yes | | 1.886 | 5.72% | No Specific Recommendation |
| Pentostatin | | Yes | | 1.886 | 5.72% | No Specific Recommendation |
| Folic Acid | | Yes | | 2 | 0.00% | No Specific Recommendation |
| Vinblastine | | Yes | | 2 | 0.00% | No Specific Recommendation |
| Carfilzomib | | Not Required | | 0.745 | 62.73% | No Specific Recommendation |
| DOPamine Hydrochloride | | Not Required | | 0.745 | 62.73% | No Specific Recommendation |
| Pemetrexed | | Not Required | | 0.764 | 61.81% | No Specific Recommendation |
| Ifosfamide | | Not Required | | 1.118 | 44.10% | No Specific Recommendation |
| Insulin Regular | | Not Required | | 1.213 | 39.33% | No Specific Recommendation |
| Polatuzumab | | Not Required | | 1.213 | 39.33% | No Specific Recommendation |
| Daratumumab | | Not Required | | 1.491 | 25.46% | No Specific Recommendation |
| Daunorubicin liposomal | | Not Required | | 1.491 | 25.46% | No Specific Recommendation |
| Dexamethasone | | Not Required | | 1.491 | 25.46% | No Specific Recommendation |
| Etoposide | | Not Required | | 1.491 | 25.46% | No Specific Recommendation |
| Cyclophosphamide | | Not Required | | 1.528 | 23.62% | No Specific Recommendation |
| Dinutuximab | | Not Required | | 1.795 | 10.25% | No Specific Recommendation |
| Melphalan | | Not Required | | 1.795 | 10.25% | No Specific Recommendation |
| Nitroglycerin | | Not Required | | 1.795 | 10.25% | No Specific Recommendation |
| Paclitaxel | | Not Required | | 1.886 | 5.72% | No Specific Recommendation |
| Gemtuzumab ozogamicin | | Not Required | | 2 | 0.00% | No Specific Recommendation |
